# Discovery of Novel GR Ligands toward Druggable GR Antagonist Conformations Identified by MD Simulations and Markov State Model Analysis

**DOI:** 10.1002/advs.202102435

**Published:** 2021-11-26

**Authors:** Xueping Hu, Jinping Pang, Jintu Zhang, Chao Shen, Xin Chai, Ercheng Wang, Haiyi Chen, Xuwen Wang, Mojie Duan, Weitao Fu, Lei Xu, Yu Kang, Dan Li, Hongguang Xia, Tingjun Hou

**Affiliations:** ^1^ Innovation Institute for Artificial Intelligence in Medicine of Zhejiang University College of Pharmaceutical Sciences Zhejiang University Hangzhou Zhejiang 310058 China; ^2^ State Key Lab of CAD&CG Zhejiang University Hangzhou Zhejiang 310058 China; ^3^ Key Laboratory of magnetic Resonance in Biological Systems State Key Laboratory of Magnetic Resonance and Atomic and Molecular Physics National Center for Magnetic Resonance in Wuhan Wuhan Institute of Physics and Mathematics Chinese Academy of Sciences Wuhan Hubei 430071 China; ^4^ Institute of Bioinformatics and Medical Engineering School of Electrical and Information Engineering Jiangsu University of Technology Changzhou 213001 China; ^5^ Department of Biochemistry and Research Center of Clinical Pharmacy of The First Affiliated Hospital Zhejiang University School of Medicine Hangzhou Zhejiang 310058 China

**Keywords:** glucocorticoid, ligand binding domain, Markov state model, nuclear receptor, virtual screening

## Abstract

Binding of different ligands to glucocorticoid receptor (GR) may induce different conformational changes and even trigger completely opposite biological functions. To understand the allosteric communication within the GR ligand binding domain, the folding pathway of helix 12 (H12) induced by the binding of the agonist dexamethasone (DEX), antagonist RU486, and modulator AZD9567 are explored by molecular dynamics simulations and Markov state model analysis. The ligands can regulate the volume of the activation function‐2 through the residues Phe737 and Gln738. Without ligand or with agonist binding, H12 swings from inward to outward to visit different folding positions. However, the binding of RU486 or AZD9567 perturbs the structural state, and the passive antagonist state appears more stable. Structure‐based virtual screening and in vitro bioassays are used to discover novel GR ligands that bias the conformation equilibria toward the passive antagonist state. HP‐19 exhibits the best anti‐inflammatory activity (IC_50_ = 0.041 ± 0.011 µm) in nuclear factor‐kappa B signaling pathway, which is comparable to that of DEX. HP‐19 also does not induce adverse effect‐related transactivation functions of GR. The novel ligands discovered here may serve as promising starting points for the development of GR modulators.

## Graphical abstract

The glucocorticoid receptor (GR) ligand binding domain structural ensemble is characterized by the molecular dynamics (MD) simulations and Markov state model analysis. Based on the GR antagonist conformations sampled by MD simulations, virtual screening and in vitro bioassays are performed. Six novel GR ligands with anti‐inflammatory activities are discovered. HP‐19 is a promising candidate for the development of GR modulators.

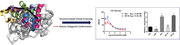

## 1 Introduction

Glucocorticoids (GCs) are a class of endogenous steroid hormones that participate in a variety of physiological functions, such as metabolism, growth, development behavior, and apoptosis.^[^ ^1^ ^]^ Since their introduction in the late 1940s, GCs such as dexamethasone (DEX) have been widely used to treat a wide variety of inflammatory, allergic, and immunologic disorders.^[^ ^2^ ^]^ However, the therapeutic use of GCs is limited by an extensive range of adverse effects, such as osteoporosis, hyperglycemia, and hypertension.^[^ ^3^ ^]^ Several selective glucocorticoid receptor (GR) modulators with reduced side effects have been developed,^[^ ^4^ ^]^ such as CpdA^[^ ^5^ ^]^ and Mapracorat.^[^ ^6^ ^]^ However, there is limited clinical progress for those compounds, and the need for safer GR modulators remains.

**Figure 1:**
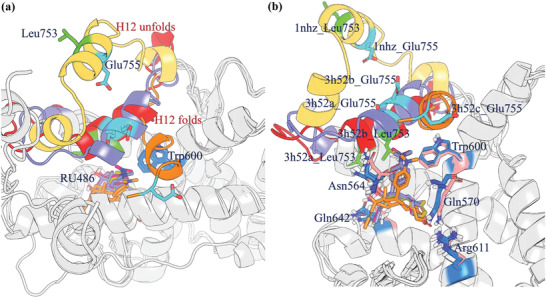
Superimposition of the crystal structure of the GR LBD in the presence of antagonists. a) The structural changes of the H12 and H11‐H12 loops (residues 741–766) in the unfolded (PDB ID: 1NHZ, yellow), active (PDB ID: 3H52_A, chain a, red), partial active agonist/antagonist (PDB ID: 3H52_B, chain b, violet), and passive (PDB ID: 3H52_C, chain c, orange) antagonist conformations. H12 is located on the opposite side of the dimethyl amino group of RU486 in 3H52_A and 3H52_C. In 3H52_C, H12 is shortened by two helix turns, in which Glu755 covers the H12 N‐terminus, and Leu753 is no longer a part of H12. b) The binding mode of RU486 in the four GR LBD antagonist conformations. The residues Gln570, Asn564, Trp600, Arg611, and Gln642 remain virtually unchanged in the active (in blue) and passive (in pink) conformations. Leu753, which faces the steroid cavity in the active conformations, was not resolved in 3H52_C.

GCs exert their physiological and therapeutic effects by binding to GR,^[^ ^7^ ^]^ which is a member of the nuclear receptor (NR) superfamily of ligand‐dependent transcription factors. Similar to most NRs, GR is organized into four major domains, including an N‐terminal activation function‐1 domain, a DNA binding domain, a hinge region, and a C‐terminal ligand binding domain (LBD).^[^ ^8^ ^]^ Analysis of the crystal structures of the GR LBD in complex with different ligands has illustrated that the conformations of the GR LBD are tightly regulated by ligand binding.^[^ ^9^ ^]^ In the structure of the agonist‐GR complex, the LBD folds into a canonical three‐layer helical sandwich, and the C‐terminal activation function‐2 (AF2) helix is stabilized in the active conformation that forms a charge‐clamp pocket to facilitate the binding of coactivators.^[^ ^10^ ^]^ While in the structure of the GR‐antagonist complex, the region between the end of helix 11 (H11) and the end of helix 12 (H12) is very flexible (**Figure** **1**). According to the study reported by Kauppi et al.,^[^ ^11^ ^]^ H12 in the crystal structures of GR in complex with the antagonist mifepristone (RU486) solved in several different crystal forms would adopt the active antagonist (named 3H52_A), partial agonist/antagonist (named 3H52_B), and passive antagonist (named 3H52_C) conformations.^[^ ^11^ ^]^ In 2003, Kauppi et al. also resolved a crystal structure of the RU486‐GR complex, in which the dimethylaniline group of RU486 appears to prevent the H12 folding, and H12 together with the former end of H11 stretch out.^[^ ^12^ ^]^ However, the static crystal structures cannot provide enough information to fully understand the dynamic process of these conformational changes. A better understanding of these structural details should contribute to the design and discovery of more effective GR ligands with improved safety profiles.

It is believed that transactivation is responsible for most adverse effects of GCs while “tethered indirect transrepression” predominantly mediates their anti‐inflammatory effects.^[^ ^13^ ^]^ Selective GR modulators are supposed to tend to induce certain receptor conformations that facilitate the interactions of monomeric GR with other transcription factors, such as nuclear factor‐kappa B (NF‐*κ*B)^[^ ^14^ ^]^ and activator protein‐1 (AP‐1),^[^ ^15^ ^]^ thereby producing tethered indirect transrepression.^[^ ^13^, ^16^ ^]^ AZD9567 is a potent GR modulator discovered by AstraZeneca,^[^ ^17^ ^]^ which can effectively alleviate the disease symptoms in rheumatoid arthritis (NCT03368235). AZD9567 appears to be safe and well tolerated in healthy population.^[^ ^18^ ^]^ And the safety profile of AZD9567 is being further evaluated in adults with type 2 diabetes (NCT04556760). A study on AZD9567 shows that it behaves as an antagonist (56% efficacy) in the transactivation assay in antagonist mode (TA_antag_) and a partial agonist (36% efficacy) in the transactivation assay in agonist mode (TA_ag_), and has 87% efficacy in the tethered indirect transrepression assay.^[^ ^17^ ^]^ However, the X‐ray structure of the GR LBD in complex with AZD9567 (PDB ID: 6EL9) is similar to the agonist conformation of GR (PDB ID: 1M2Z). This structure cannot clarify how the binding of AZD9567 perturbs the structural state of the receptor and also cannot explain why AZD9567 can exhibit antagonist activity and inhibit the DEX response in reporter gene assays.

Molecular dynamics (MD) simulation has been widely used to investigate the conformational dynamics and protein‐ligand interaction of NRs.^[^ ^19^ ^]^ In 2020, Alves et al. employed steered MD simulations (SMD) and umbrella sampling to explore the transitions between the GR antagonist conformation and agonist conformation.^[^ ^20^ ^]^ They found that upon the binding of the antagonist RU486, H12 occupies the AF2 groove and the passive antagonist conformation is the lowest‐energy state, while in the agonist conformation the agonist DEX anchors H12. However, in their study, an external force was applied to drive the system along the transition from the GR passive antagonist conformation to the GR active antagonist conformation. Therefore, the conformational spaces of H12 sampled by SMD in different complexes were similar, and how the ligands affect the H12 conformational space was not well depicted.

In this study, we carried out the all‐atom MD simulations and Markov state model (MSM) analysis to explore the conformational dynamics of the LBD and the complex of LBD bound with the agonist DEX, antagonist RU486, and modulator AZD9567. It's observed that upon the binding of the agonist DEX, H12 shifts away from its passive antagonist position, and stabilizes in the active antagonist state. Whereas binding of RU486 or AZD9567 changes the folding pathway of H12, and the passive antagonist state appears more stable. Therefore, it can be deduced that if a ligand can bias the conformation equilibria toward the passive antagonist state, it probably exhibits reduced side effects. To prove this hypothesis, we carried out the structure‐based virtual screening(SBVS) based on the passive antagonist conformation, producing a total of 88 potential ligands. Then the compounds were submitted for bioassays, and six of them that can indeed target the ligand binding pocket (LBP) of GR exhibit good bioactivities. Therein, compound HP‐19 can inhibit NF‐*κ*B signaling (IC_50_ = 0.041 ± 0.011 µm) while does not influence GR transactivation. Our study provides valuable clues for the development of potent GR modulators with novel scaffolds and improved safety profiles.

## 2 Results and Discussion

### 2.1 The Binding of Different Ligands Induces Multiple Intermediate States of GR H12

The interactions between the GR LBD and its coactivators or corepressors are quite sensitive to the conformations of the GR LBD induced by the binding of different GR ligands.^[^ ^21^ ^]^ There is a dynamic communication network between the LBP and AF2, and the GR H12 exhibits ligand‐ and coregulator‐dependent dynamics.^[^ ^22^ ^]^ Recent studies on another NR, the peroxisome proliferator‐activated receptor gamma, have demonstrated that the relative population of the H12 conformations would respond to ligand binding.^[^ ^23^ ^]^ However, we still have very limited knowledge about the structural ensembles of the GR H12 and how the binding of pharmacologically distinct ligands affects the conformational ensemble of the coregulator‐binding surface and H12. Therefore, in order to generate the reliable structural ensembles of the GR LBD, extensive MD simulations were carried out to the LBD without any ligand (apo‐LBD) and the complexes of LBD bound with the agonist DEX (dex‐LBD), antagonist RU486 (ru486‐LBD), and modulator AZD9567 (azd‐LBD) starting from the same unfolded H12 conformation (Figure S1, Supporting Information).

**Figure 2:**
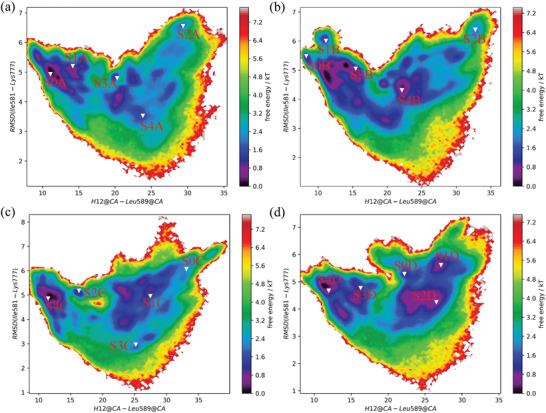
The free energy landscapes based on the RMSD of Ile581‐Lys777 of the GR LBD and the center‐of‐mass distance between the H12 (Ala754‐Tyr764) and Leu589 (a) apo‐LBD; b) dex‐LBD; c) azd‐LBD; d) ru486‐LBD). The representative structures for the five states (S0, S1, S2, S3, and S4) are shown in the free energy landscapes.

**Figure 3:**
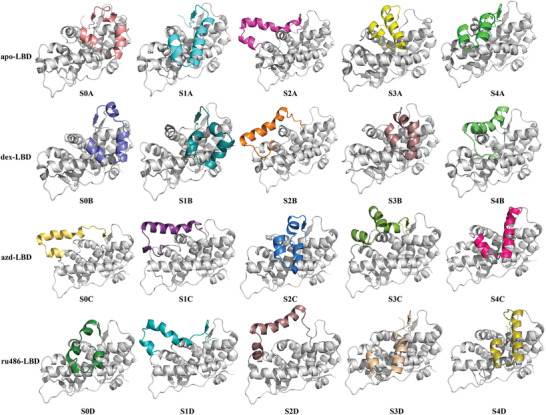
Representative structures of the five metastable macrostates calculated by PCCA in apo‐LBD, dex‐LBD, azd‐LBD, and ru486‐LBD.

According to the static structures, it can be observed that GR agonists, such as DEX, can induce the conformational shift of H12 to form a part of AF2, while in the passive antagonist conformation (3H52_C), H12 folds into the position of AF2, preventing the binding of coactivators. The rotation of H12 is a hallmark of the GR activation. Hence, we characterized the conformational changes of the AF2 of GR by measuring the distance between H12 (Ala754‐Tyr764) and Leu589 (named H12‐Leu589). The root‐mean‐square deviation (RMSD) of the C_*α*_ atoms of Ile581‐Lys777 for each system relative to the initial coordinates were used to characterize the conformational changes of the GR LBD. The 2D free energy landscapes as a function of the RMSD of Ile581‐Lys777 of the GR LBD and the distance H12‐Leu589 were calculated (**Figure** **2**). Five metastable conformations were identified and denoted as S0, S1, S2, S3, and S4 (Figure S2, Supporting Information). In addition, the perron cluster cluster analysis (PCCA) distributions in PyEMMA were used to select the representative structures of the 5 metastable macrostates (S0–S4, **Figures** 2,**3**).

The AF2 surface of apo‐LBD shows considerable conformational changes. with a wide H12‐Leu589 distance distribution (7.6 to 35 Å, Figure 2a). Helix 3 (H3) and helix 4 (H4) of the GR LBD are relatively stable, and the main conformational changes take place on H12 (Figure 3). H12 possesses multiple conformational states, and it can shift inward or outward. The S0A and S1A states of apo‐LBD (Figure 3) are intermediates that are close to the passive antagonist state (3H52_C). Moreover, a structural ensemble of the H12 folding outside of H11 was observed (S2A, Figure 3). The representative structure of S3A is close to the agonist state (1M2Z). The representative structure of S4A is close to the active antagonist state (3H52_A). To detect the sampling of the experimental AF2 conformations by the MD simulations, the RMSD of the AF2 C_*α*_ atoms (including the residues 567–579, 590–597, and 754–764) was used as the criterion to compare the similarity between the conformations from the 10 µs MD simulation trajectories and the experimental structure. The probability distribution of the AF2 RMSD shows that the inactive states (including the partial agonist/antagonist state (3H52_B, 2%) and passive antagonist state (3H52_C, 2%), Table S1, Figure S3, Supporting Information) are highly populated, while the active states (including the active antagonist state (3H52_A, ≈0.0%) and agonist state (1M2Z, 0%), Table S1, Figure S3, Supporting Information) are lowly populated. In conclusion, the physiologic structural ensemble of the GR H12 is very diverse. Without ligand binding, the GR LBD can only sample to the 3H52_B and 3H52_C states.

In contrast, although the conformational space of dex‐LBD is similar to that of apo‐LBD (Figure 2b), the populations of the active antagonist state (3H52_A) and agonist state (1M2Z) are increased (Table S1, Figure S3, Supporting Information). It has been reported that the binding of DEX can trigger the recruitment of coactivators to GR, and then GR is activated for transactivation or also possibly for transrepression,^[^ ^24^ ^]^ which is associated with not only the therapeutic efficacy but also the adverse effects of GCs.^[^ ^25^ ^]^ Therefore, the active antagonist state (3H52_A) and agonist state (1M2Z) may be related to both of therapeutic and adverse effects. Compared with the dex‐LBD system (7.27 to 36.56 Å), azd‐LBD undergoes more significant conformational changes, and the distribution of H12‐Leu589 is wider (8.37 to 40.00 Å), indicating a higher flexibility of the azd‐LBD H12. In addition, the S0C and S1C states of azd‐LBD are different from dex‐LBD and apo‐LBD whose H12 fold outside of H11 (Figure 3). The representative structures of S2C and S4C in azd‐LBD are close to the passive antagonist state (3H52_C). In the S3C state of azd‐LBD, H12 folds to the inside of H11 (Figure 3), exhibiting a specific conformational state. Nevertheless, the state is different from the agonist state and close to the active antagonist state, possibly supporting the partial activity of AZD9567 in the transactivation assay.^[^ ^17^ ^]^

The conformational landscape of ru486‐LBD is quite special (Figure 2). As shown in Figure 3, in the S0D state of ru486‐LBD, a part of H11 folds into the AF2 site, preventing the binding of coactivators. The dimethyl amino phenyl moiety of RU486 clashes with H12, and the conformation of H12 should be adjusted. However, the isopropyl group of AZD9567 is smaller, and it may not exert a substantial influence on the conformation of H12. In the S1D and S2D states, H12 locates outside of H11, which are similar to the S1C state of azd‐LBD. The representative structure of S3D in ru486‐LBD is close to the passive antagonist state (3H52_C). In addition, as shown in Table S1 and Figure S3, Supporting Information, in the collectable samples of the ru486‐LBD system, the active antagonist state (3H52_A) is lowly populated (≈0.0), and the passive antagonist state is highly sampled (3h52_C, 6%), suggesting that a single conformation of H12 dominates its conformational ensemble for the ru486‐LBD complex. That is to say, upon the binding of RU486, H12 of the GR LBD is mainly induced to dock into the AF2 site, resulting in the occupation of the site, thus preventing coactivator binding. Compared with DEX and AZD9567, RU486 shows neither their anti‐inflammatory activities nor their adverse effects, so the populations of the passive antagonist state (3h52_C) are probably unrelated to anti‐inflammatory or adverse effects.

In summary, with or without ligand binding, the GR LBDs are sampled in variant conformational spaces (Figures 2, 3). It has been reported that GR H12 exhibits both the ligand‐ and coregulator‐dependent kinetics.^[^ ^22^ ^]^ However, we found that the ligand alone can affect the conformation of H12, although only a 1–2% change. The binding of the GR agonists (DEX and AZD9567) induces more active antagonist state (3H52_A) and agonist state (1M2Z), and the antagonist binding mainly shifts the populations toward the inactive state (3H52_C).

### 2.2 Phe737 and Gln738 are Key Residues for Sending Message from Ligands to AF2

**Figure 4:**
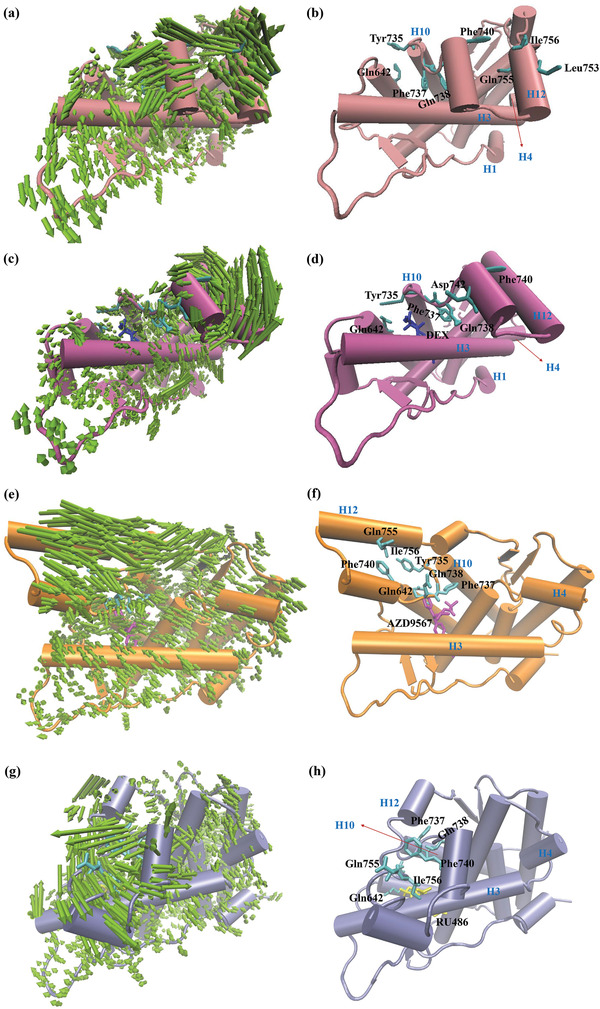
Representation of the PC1 and the interaction patterns between the ligand binding site as well as H12 and different ligands: a,b) GR without ligand binding, c,d) GR binding with DEX, e,f) GR binding with AZD9567, and g,h) GR binding with RU486. The heavy atoms of the GR‐LBDs were used in the calculations. The lengths of the green arrows are correlated to the scope of the backbone movements. AF2 was a majorly hydrophobic groove formed by residues from H3, H4, and H12.

To discover the details about how the ligand interacts with H12 of the GR LBD in each system, principal component analysis (PCA) was performed on the conformations taken from the representative trajectory based on the coordinates of all the heavy atoms, in which H12 folded into a conformation close to the S0 states. The schematic representation of the first principal component (PC1) for each system is shown in **Figure** **4**. For apo‐LBD, dex‐LBD, azd‐LBD, and ru486‐LBD, PC1 dominates around 55.0%, 67.3%, 63.16%, and 47.23% of the variance, respectively. As shown in Figure 4, as to the binding of the agonist, antagonist, or modulator, the conformational fluctuations of H12 are different. Without ligand binding or upon agonist binding, H12 moves outward and the volume of AF2 tends to increase (Figure 4a,c). While the binding of the antagonist (RU486) or modulator (AZD9567) would change the swing direction of H12 from outward to inward (Figure 4e,g). And in all systems, the fluctuations of the regions around the N‐terminal of H12 and the C‐terminal of H11 are larger than those around the C‐terminal of H12, indicating that the residues at the N‐terminus of H12 and the C‐terminus of H11 are probably the key amino acids for the communications between ligands and AF2.

Further analysis shows that without ligand binding, the hydrophobic LBP formed by Tyr735, Gln642, Gln755, Ile756, and Phe740 are occupied by Phe737 and Gln738 (Figure 4b). Upon agonist binding, a hydrogen bond is formed between Glu642 and DEX, and then Phe737 and Gln738 rotate away from DEX, but still participate in the formation of the hydrophobic pocket. Meanwhile Asp742 rotates to the ligand to help the formation of the hydrophobic pocket, which in turn causes H12 to move out of AF2 (Figure 4d). While upon the binding of AZD9567 and RU486 (Figure 4f,h), the binding site is reorganized. The dimethyl amino phenyl moiety of RU486 and the isopropyl group of AZD9567 bind to the original position of Phe737. The space conflict between the ligands and the aromatic ring of Phe737 destabilizes the H11 C‐terminal, and as a result, Gln738, Phe740, Glu755, and Ile756 rotate away from the ligands, accounting for the inward movement of H12. In conclusion, the residues Phe737 and Gln738 play critical roles in the mediation of the interactions between the ligands and AF2. The antagonist and modulator are in steric clash with H12, causing H12 to move inward. Therefore, the volume of AF2 tends to decrease, and the possibility of the binding with coactivators also decreases. In contrast, there is no steric clash between the agonist and H12, and H12 can stay outward to form a transcriptionally active conformation.

### 2.3 GR Modulator and Antagonist Change the Folding Pathway of H12

**Figure 5:**
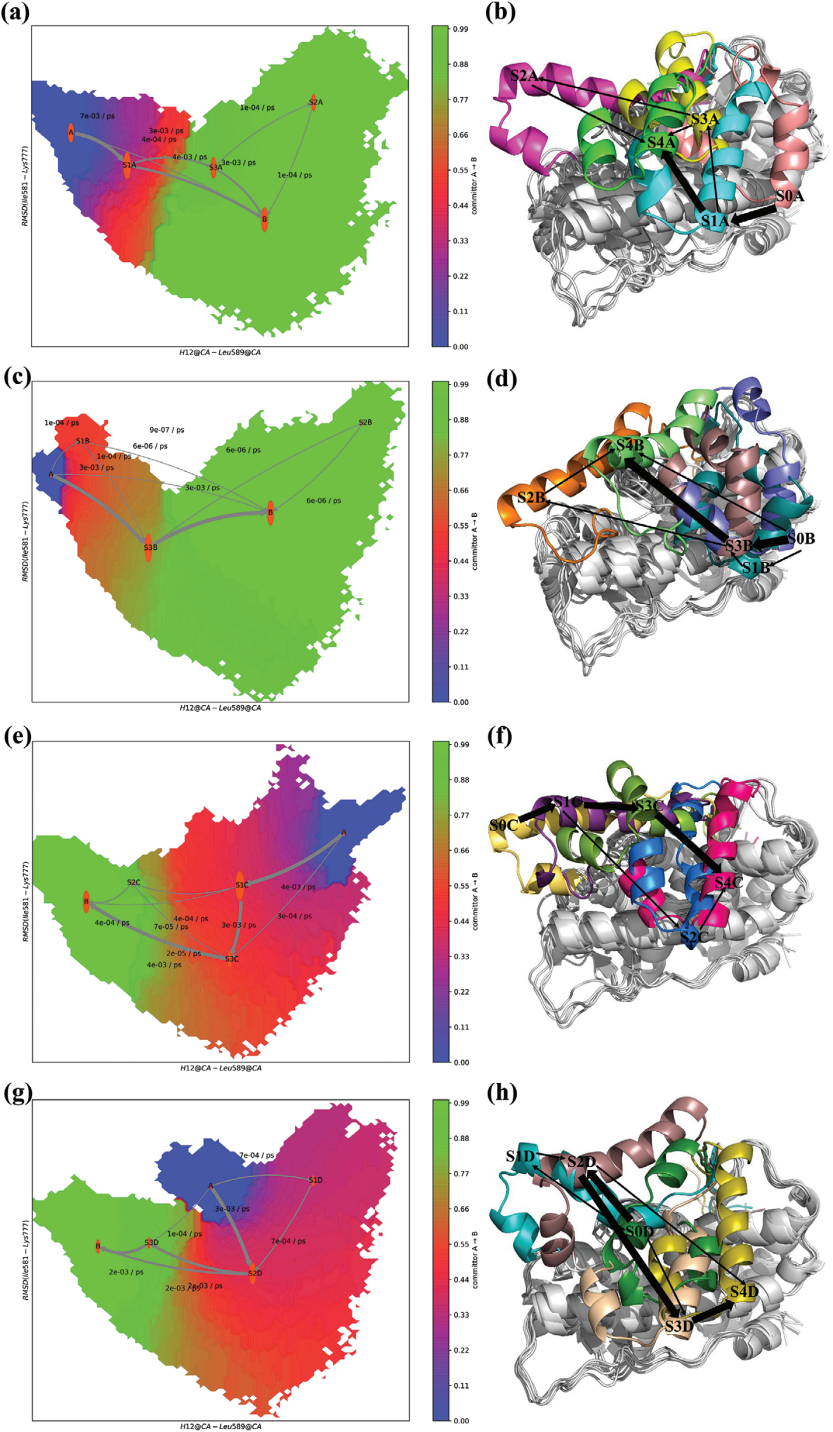
Conformational conversion pathway of H12. a) The transformation pathway network in apo‐LBD. The weights of the arrows indicate the transition probability between states, and labels upon the arrows denote the proportion of flux in the current segment. The size of each state is proportional to the life time of the corresponding state. b) Structures of the main pathways in apo‐LBD. c) The transformation pathway network in dex‐LBD. d) Structures of the main pathways in dex‐LBD. e) The transformation pathway network in azd‐LBD. f) Structures of the main pathways in azd‐LBD. g) The transformation pathway network in ru486‐LBD. h) Structures of the main pathways in ru486‐LBD.

To elucidate the mechanism of the conformational changes of H12, the MSM analysis was performed to build the kinetic community networks for the studied systems, which helps to identify the key conformational states of the GR LBD and quantify the state thermodynamic populations and the kinetics of state transitions. The transition matrices with different values of the lag time *τ* were computed. At *τ* = 0.2 ns, these timescales almost converged (Figure S4, Supporting Information). Thus, the lag time was set to *τ* = 0.2 ns. The Chapman–Kolmogorov test (CK test) for the eight microstates was conducted to evaluate the validity of the MSM. As shown in Figures S5–S8, a nearly perfect agreement could be achieved between the estimated transition probabilities calculated from the MD data (circles) and the predictions of the MSMs for all lag times (0.2, 0.4, 0.6, and 0.8 ns), highlighting the high Markovianity of the microstates. The fluxes and pathways were computed by the transition path theory (TPT). The total flux from the initial state A (S0) to the target state B (S4) was decomposed into pathways (**Figure** **5**, **Table** **1**; Figure S9, Supporting Information).

**Table 1:** Pathways induced by different ligands from the MSM analysis

Ligand	Pathways	Percentage of total coarse flux [%]
apo	S0A→S1A→S4A	58.0
	S0A→S1A→S3A→S4A	34.6
	S0A→S4A	5.6
	S0A→S1A→S3A→S2A→S4A	1.8
DEX	S0B→S3B→S4B	96.0
	S0B→S1B→S3B→S4B	3.6
	S0B→S4B	0.2
	S0B→S1B→S3B→S2B→S4B	0.2
AZD9567	S0C→S1C→S3C→S4C	81.7
	S0C→S1C→S2C→S4C	9.4
	S0C→S3C→S4C	6.6
	S0C→S1C→S4C	1.8
	S0C→S3C→S2C→S4C	0.5
RU486	S0D→S2D→S3D→S4D	55.3
	S0D→S2D→S4D	23.9
	S0D→S1D→S2D→S4D	17.5
	S0D→S3D→S4D	3.4

Without ligand binding, the agonist state (1M2Z) was an unstable intermediate state and cannot be captured (population = 0, Table S1, Supporting Information). The positions of H12 mainly changed from S0A to S4A through the S1A state (occupying 58.0% of the total flux) (Figure 5b; Figure S9, Supporting Information). Another pathway, in which H12 went through the lowly populated S3A state, was possible but less frequent (occupying 34.6% of the total flux). Upon the binding of DEX, the conformational conversion pathway of H12 was similar to that in apo‐LBD (Figure 5c,d), and H12 rotated away from Leu589 in H4 to visit different folding positions (occupying 96.0% of the total flux). But the S3B state became more populated than that in apo‐LBD. This implies that although the steric conflict between the agonist and Phe737 leads to the state transitions of H12, the interaction between the agonist and Leu753 in H12 can stabilize the agonist state.^[^ ^10^ ^]^

Upon the binding of AZD9567, H12 rotates close to Leu589 from the S0C state, which is different from that in apo‐LBD and dex‐LBD. In the azd‐LBD system, the folding pathway for H12 with the largest flux is S0C‐S1C‐S3C‐S4C, which occupies 81.7% of the total flux (Table 1, Figure 5e). The atomistic picture of the conformational changes is shown in Figure 5f and Figure S9, Supporting Information. S4C is the most stable state, which is close to the passive antagonist state (3H52_C). The states S1C and S3C are the intermediate states, and they can easily relax to S4C (Figure 5e). In this case the passive antagonist state (S2C of azd‐LBD) is more stable than that in dex‐LBD and apo‐LBD, leading to a decreased possibility for the coactivator binding, which was conducive to both anti‐inflammatory activity and adverse effects. This is consistent with the reported fact that AZD9567 can cause some mild or moderate intensity of adverse events.^[^ ^18^ ^]^

Upon the binding of the antagonist RU486, the positions of H12 mainly change from S0D to S4D through the S2D and S3D states (occupying 55.3% of the total flux, Figure 5g,h). The clusters S0D and S3D are both the passive states. And the 3H52_A state is a lowly populated (≈0.0). This may be because the dimethyl amino phenyl moiety of RU486 conflicts with Phe737 and Leu753 at the same time, and the full antagonist cannot stabilize the active conformation without co‐regulatory factors. Therefore, the binding of an antagonist can enhance the probability for the passive antagonist state, resulting in a much lower probability of the downstream binding of coactivators. This may be one of the reasons why the full antagonists significantly reduce the side effects.^[^ ^26^ ^]^

Based on the above results, it is proposed that the GR modulator and antagonist can perturb the structural state of the GR LBD and change the folding pathway of H12. The compounds which can bias the conformational equilibria toward the passive antagonist state would be better in reducing adverse effects and balancing the therapeutic effect of GC.

### 2.4 Discovery of Novel GR Modulators Based on Passive Antagonist Conformation

It has become widely accepted that the transcription activation by GR contributes to the majority of side effects. Therefore, if a compound can properly induce passive antagonist conformations, it may exhibit reduced side effects and at the same time still keep anti‐inflammatory activity.

**Figure 6:**
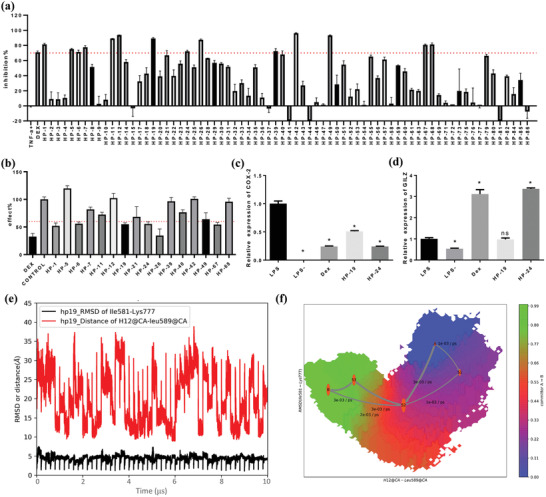
In vitro bioassays of the potential hits. a) Tethered indirect transrepression activities of the 72 compounds at the concentration of 10 µm (*n* = 3 per group). b) GR binding activities of the 16 compounds at the concentration of 10 µm (*n* = 4 per group). c,d) qPCR analysis of the effect of the DEX, HP‐19, and HP‐24 treatment on the mRNA expression levels of COX‐2 and GILZ (data presented as mean ± SEM, *n* = 6, *p*‐values are calculated using one‐way ANOVA followed by Dunnett's post hoc test, *p < 0.05). e) The evolution of the RMSD of Ile581‐Lys777, and the distance between H12 (Ala754‐Tyr764) and Leu589 over time in HP19‐LBD. f) The transformation pathway network in HP19‐LBD.

However, due to the flexibility of H12, the complete passive antagonist structures of the GR LBD have not yet been reported. In our study, the MD/MSM simulations revealed a range of conformational substates of the GR LBD, and the passive antagonist conformations of RU486 and AZD9567 were sampled (Table S1, Figure S3, Supporting Information). Considering the aim of discovering novel GR modulators, the Chemdiv library was virtually screened based on the passive antagonist conformation induced by AZD9567. In the passive antagonist state (Figure S10, Supporting Information), the amide of AZD9567 rotates around 90° to reduce its conflict with H11. And the isopropyl of AZD9567 forms stable hydrophobic interaction with Phe737 and Trp600. The compounds in Chemdiv were docked into the AZD9567 binding site in the passive antagonist conformation (Figure S10, Supporting Information). The top‐ranked 5000 compounds were retained as a focused library of potential GR modulators. After druglike analysis, structural clustering and visual inspection of the docked poses, a total of 88 compounds were purchased for bioactivity evaluation (Table S2, Supporting Information). At first, the cell cytotoxicity assay was performed to rule out the inherent toxicity of the tested compounds. As shown in Figure S11, Supporting Information, 72 compounds do not affect Raw264.7 cell viability at 25 µm. Subsequently, these compounds were tested by the in vitro GR ligand binding assay and transrepression assay. As shown in **Figure** **6**a, 16 compounds at 10 µm exhibit good inhibition activities (>70%) in the reporter gene assay measuring transrepression. Among the 16 compounds, six compounds exhibit specific GR binding activity (effect <60%, Figure 6b).

As shown in **Table** **2**, HP‐1 and HP‐6 behave as partial antagonists with 48.9 ± 2.41% or 30.8 ± 3.27% efficacy in the TA_antag_ assay, less than 1% efficacy in the TA_ag_ assay, and relatively lower effect in the tethered indirect transrepression assay. While compound HP‐19 exhibits very good potency in the tethered indirect transrepression assay, and it is capable of inhibiting NF‐*κ*B signaling with IC_50_ = 0.041 ± 0.011 µm (IC_50DEX_ = 0.012 ± 0.001 µm, and IC_50AZD9567_ = 0.37 ± 0.075 µm) and inhibiting AP‐1 signaling with IC_50_ = 0.79 ± 0.26 µm (IC_50DEX_ = 0.004 ± 0.001 µm, and IC_50AZD9567_ = 0.14 ± 0.04 µm). Furthermore, HP‐19 shows about 50–60% as effective as DEX for the transcription inhibition of proinflammatory enzyme cyclooxygenase‐2 (COX‐2) in the cell line of macrophage Raw264.7 (Figure 6c). In addition, HP‐19 binds to the GR LBP in a dose‐dependent manner (IC_50_ = 1.57 ± 0.71 µm), indicating that the compound can directly target the GR LBP. Besides its good anti‐inflammatory activity, the most interesting thing is that HP‐19 has little or no effect on the agonist/antagonist profile. HP‐19 not only fails to induce transactivation activity (TA_ag_, −2.1 ± 0.63%), but also cannot inhibit the DEX‐dependent transactivation (TA_antag_, 1.92 ± 0.15%). As an evidence, HP‐19 does not affect the DEX‐induced transcription of the (+) GRE transactivated glucocorticoid‐induced leucine zipper (GILZ) (Figure 6d). Taken together, HP‐19, which does not induce the transactivation functions of GR while still induces its tethered indirect transrepression activity, is a novel and potent GR modulator.

**Table 2:** The results of the reporter gene assays for the hit compounds 
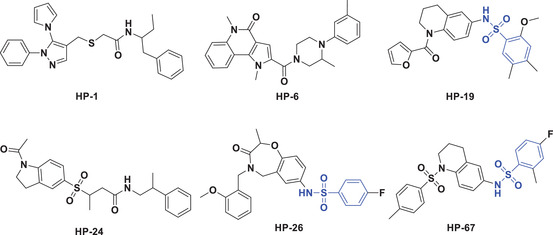

Compounds	GR	Cell transrepression		Cell transactivation	
	TR‐FRET (IC_50_ µm)	NF‐*κ*b (IC_50_ µm)	AP‐1 (IC_50_ µm)	TA_ag_ (effect%)^a)^	TA_antag_ (inhibition%)^b)^
HP‐1	2.17 ± 0.47	7.24 ± 0.25	8.45 ± 0.89	0.35 ± 0.36%@2 µm	48.9 ± 2.41%%@15 µm
HP‐6	1.42 ± 0.08	10.03 ± 1.90	5.00 ± 0.23	0.46 ± 0.41%@2 µm	30.8 ± 3.27%@15 µm
HP‐19	1.57 ± 0.71	0.041 ± 0.011	0.79 ± 0.26	−2.1 ± 0.63%@2 µm	1.92 ± 0.15%@15 µm
HP‐24	2.53 ± 0.12	2.71 ± 0.72	1.16 ± 0.35	43.2 ± 1.03%@2 µm	3.14 ± 1.34%@15 µm
HP‐26	4.45 ± 0.36	12.23 ± 1.95	5.63 ± 0.96	−0.61 ± 0.23%@2 µm	20.7 ± 2.29%@15 µm
HP‐67	1.57 ± 0.54	2.76 ± 0.18	1.56 ± 0.31	−1.77 ± 0.58%@2 µm	70.8 ± 2.01%@15 µm
DEX	0.010 ± 0.005	0.012 ± 0.001	0.004 ± 0.001	108 ± 6.0%@2 µm	ND
AZD9567	0.004^c)^	0.37 ± 0.075	0.14 ± 0.04	36.5 ± 1.78%@2 µm	77.2 ± 0.93%@15 µm

ND: Not determined;

^a)^ Direct effect of compounds on transactivation in the Hela‐MMTV‐Luc reporter cells, and the agonist activity of DEX at 10 µm was used to define the 100%;

^b)^ Inhibitory effect of compounds on DEX transactivation in Hela‐MMTV‐Luc reporter cells, and the inhibition of RU486 at 1 µm was used to define the 100%.

^c)^ L. Ripa et al.^[17]^

Another notable compound is HP‐24. It can effectively inhibit AP‐1 signaling with IC_50_ = 1.16 ± 0.35 µm. Furthermore, the mRNA level of the proinflammatory COX‐2 is substantially downregulated by HP‐24 (Figure 6c). However, HP‐24 displays a partial agonist profile (43.2 ± 1.03%) in the transactivation assay and upregulates the mRNA level of GILZ (Figure 6d). It is similar to DEX and cannot separate tethered indirect transrepression from transactivation.

The conformational changes of H12 induced by HP‐19 were also investigated by the 10 µs MD simulations and MSM analysis (Figure 6e; Figure S12, Supporting Information). In the HP19‐LBD system, the folding pathway for H12 with the largest flux was S0‐S2‐S3‐S4, which occupies 60.8% of the total flux (Figure 6f). Binding of HP19 changed the folding pathway of H12. H12 swings from outward to inward, which is similar to that in azd‐LBD. Compounds HP‐26 and HP‐67 have the same benzenesulfonamide group as compound HP‐19, but their activities are weaker in the tethered indirect transrepression assay, which anyhow provides a basis for the structural optimization of HP‐19.

## 3 Conclusion

In this study, microsecond long MD simulations and MSM analysis were employed to explore the conformational dynamics and conformational ensemble equilibria of GRs upon the binding of different ligands. We found that the GR LBD is not in a single conformational state upon the binding of agonists or antagonists, but rather samples in different conformational ensembles. In the apo‐LBD system, 1M2Z was an intermediate state and hard to be captured. Upon the binding of an agonist, the populations of the 3H52_A and 1M2Z state were increased compared with those of apo‐LBD. The binding of an agonist also induced the inactive states of the GR LBD. While in the ru486‐LBD system, the 3H52_A state is a lowly populated intermediate state, and H12 rearranges from a passive state (S0D) to another passive state (S4D) (Figure 5h). Compared with DEX and AZD9567, RU486 has no anti‐inflammatory activity, so the populations of the 3H52_A and 1M2Z states may be related to anti‐inflammatory effects. However, the increased passive antagonist state in the ru486‐LBD systems is most likely one of the reasons why the full antagonists significantly reduce the side effects. Our unbiased simulations also found a residue interaction pathway of the ligands‐Phe737‐Gln738 in GRs. To test the druggability of the passive antagonist conformation, SBVS was employed to identify potential safer GR ligands. Six compounds with specific GR binding activities and good potencies in transrepression assays were identified. Among them, HP‐19 inhibited NF‐*κ*B signaling with IC_50_ = 0.041 ± 0.011 µm, which was comparable to that of DEX (IC_50_ = 0.012 ± 0.001 µm). Further studies on structural optimization and action mechanism of HP‐19 will benefit the development of novel GR modulators.

## 4 Experimental Section

### Preparation of Simulation Systems

The crystal structure of the human GR LBD with the unfolded H12 in complex with RU486 (ru486‐LBD) was obtained from the RCSB Protein Data Bank (PDB ID: 1NHZ).^[^ ^12^ ^]^ The mutated residues in the crystal structure were mutated back (N517D, F602S, and C638D). The missing residues of the GR LBD (Gln760‐Gly767) were added in MOE 2018. The LBD without any ligand (apo‐LBD) was obtained by removing RU486. The GR agonist DEX and modulator AZD9567 were obtained from the crystal structures of the complexes (PDB IDs: 1M2Z and 6EL9). Then, the crystal structures of 1M2Z and 6EL9 were superimposed onto that of 1NHZ, and DEX and AZD9567 were extracted and merged into 1NHZ to construct the structures of the GR LBD bound with DEX and AZD9567 (dex‐LBD and azd‐LBD). Finally, four systems (i.e., apo‐LBD, ru486‐LBD, dex‐LBD, and azd‐LBD) were prepared for the MD simulations.

The AMBER ff14SB force field^[^ ^27^ ^]^ and the general AMBER force field (GAFF2)^[^ ^28^ ^]^ were used for the proteins and ligands, respectively. The atomic charges for each small molecule were generated by fitting the electrostatic potential calculated at the Hartree‐Fock SCF/6‐31G basis set by using Gaussian^[^ ^29^ ^]^ through the restrained electrostatic potential algorithm^[^ ^30^ ^]^ implemented in the antechamber module of AMBER18.^[^ ^31^ ^]^ Each system was solvated in an octahedral TIP3P water box,^[^ ^32^ ^]^ and the solute atoms were at least 10 Å away from the boundary of the water box. The counter Na^+^ ions were added to neutralize the net charge of each system.

### MD Simulations

All simulations were performed with AMBER18.^[^ ^31^ ^]^ The Particle mesh Ewald (PME) algorithm was used to handle the long‐range electrostatic interactions,^[^ ^33^ ^]^ and the non‐bonded cutoff for the real‐space interactions was set to 12 Å. Each system was minimized by the following three‐step protocol. First, the water molecules and counter ions were optimized by 1000 steps of steepest descent and 2000 steps of conjugated gradient minimizations with the protein and ligand atoms restrained by 50 and 10 kcal mol^−1^Å^−2^, respectively. Then, the protein atoms were restrained by a 10 kcal mol^−1^Å^−2^ force constant and the other atoms were minimized by 1000 steps of steepest descent and 2000 steps of conjugated gradient minimizations. Finally, the entire system was minimized by 1000 steps of steepest descent and 2000 steps of conjugated gradient minimizations without any restraint. Next, each system was gradually heated from 100 to 300 K over a period of 30 ps in the NVT ensemble, and 110 ps equilibration was performed at 300 K. Finally, 200 ns MD simulations were carried out in the NPT (*T* = 300 K and *P* = 1 atm) ensemble with the PMEMD program.^[^ ^34^ ^]^ The SHAKE algorithm^[^ ^35^ ^]^ was used to constrain the covalent bonds involving hydrogen atoms and the time step was set to 2 fs. The snapshots were saved every 10 ps. For each system, a total of 50 independent MD simulations were conducted, and in total 40 µs trajectories were generated for the four systems (4 × 50 × 200 ns).

The RMSD, distance, and secondary structures analyses were carried out by the cpptraj module in AmberTools18. The PCA for each system was carried out by the NMWiz module in VMD^[^ ^36^ ^]^ based on the coordinates of all the heavy atoms in the trajectories.

### MSM Analysis

MSM analysis is a powerful tool to turn a group of short trajectories into a scientifically meaningful model of dynamics.^[^ ^37^ ^]^ Here, the MSM were built using the PyEMMA^[^ ^38^ ^]^ software package (version 2.5.7) through following workflows. First, each frame in the MD trajectories was transformed into a vector composed by the RMSD of Ile581‐Lys777 and the distance between H12 (Ala754‐Tyr764) and Leu589, by which different conformations could be discriminated. Second, the conformations for each system were clustered into 1000 clusters (microstates) using the *k*‐means algorithm. Third, a transition count matrix was constructed by counting the number of the transitions between each pair of microstates at a proper lag time (time interval) using the sliding window approach, and then the transition probability matrix was obtained by the Bayesian MSM estimator.^[^ ^39^ ^]^ The timescales were examined to determine the lag time when the system becomes Markovian.^[^ ^40^ ^]^ As shown in Figure S4, Supporting Information, 0.2 ns was chosen as the lag time. The CK test^[^ ^41^ ^]^ was employed to evaluate the validity of the 1000‐state Bayesian Markov model (Figures S5–S8). Seven slow processes up to the lag time of 0.8 ns could be determined. A perfect agreement was observed between the estimated transition probabilities calculated from the MD data and the predictions of the MSMs, suggesting the validity of the MSMs. After that, these 1000 microstates were further divided into macrostates using the PCCA algorithm.^[^ ^42^ ^]^ Finally, the TPT^[^ ^43^ ^]^ was used to elucidate the transitions between these macrostates and the highest‐flux pathway.

### SBVS Workflow

SBVS based on the passive antagonist conformation of azd‐LBD was expected to discover novel safer GR ligands. However, a completely folded crystal structure of GR in the passive antagonist state was never resolved. The passive antagonist conformation (PDB ID: 3H52) reported by Schoch et al.^[^ ^11^ ^]^ has a missing region (the end of H11 up to a few residues before H12, residues 741–753), and its N‐terminal end (helix 1 (H1) and H1‐H3 loop) is unfolded from the LBD body. To obtain a completely folded passive antagonist conformation, the RMSD of the AF2 C_*α*_ atoms were used as the criterion to compare the similarity between the conformation from the 10 µs MD simulation trajectories and the passive antagonist conformation reported by Schoch et al. The conformation with the smallest RMSD (RMSD = 1.47 Å) was selected for the SBVS.

The protein structure was prepared by using the Protein Preparation Wizard in Schrödinger 2019. The binding site was defined as the region centered on the center‐of‐mass of AZD9567 with the size of 10 Å × 10 Å × 10 Å using the Receptor Grid Generation component of Glide. The small molecules in the ChemDiv chemical library processed by LigPrep were docked into the prepared structure by using the Glide module, and the binding energies were scored and ranked by the Glide SP scoring mode. The 5000 top‐ranked compounds were filtered by the Lipinski's rule‐of‐five and Oprea's rules, and then the remaining molecules were clustered based on the 2D similarity (Tanimoto coefficient) of the MACCS fingerprints. Finally, 88 potential compounds were purchased for subsequent bioassays. The bioactivities and structures of the 88 compounds are listed in Table S2, Supporting Information.

### Materials for Bioassays

Hela (CLS Cat# 300194/p772_HeLa) and RAW264.7 (CLS Cat# 400319/p462_RAW‐2647) (a murine macrophage cell line) cells were grown in high‐glucose DMEM supplemented with glutamine, penicillin, streptomycin, and 10% FBS (Gibco). pCMV‐GR11 (Addgene, #89105) was a gift from Elizabeth Wilson. pGL4.36[luc2P MMTV Hygro] and NF‐*κ*B‐Luc were purchased from Promega and Beyotime, respectively. Five copies of the AP‐1 promoter were cloned into the BmtI and BglII sites of pNF‐*κ*B‐luc. LPS was from Escherichia coli O55:B5 (Sigma Aldrich; L‐2637). Recombinant human tumor necrosis factor‐alpha (TNF‐*α*) was purchased from Sangon Biotech. Phorbol myristate acetate (PMA), AZD9567, and DEX were purchased from MedChemExpress (MCE), and all the tested compounds were bought from TargetMol.

### Cytotoxicity Assay

For cytotoxicity assay, Raw264.7 cells were used to rule out the inherent toxicity of the tested compounds. Raw264.7 cells were cultured in DMEM media at a density of 8 × 10^4^ cells per well and then treated with the tested compounds at the concentration of 25 µm for 48 h. 10 µL of 5 mg mL^−1^ MTT solution was added into each well and incubated for 3 h, then 100 µL of triplex solution (10% SDS, 5% isobutyl alcohol, and 0.012 mol L^−1^ HCl) was added to dissolve the formazan crystals. Absorbance was measured at 540 and 630 nm with a spectrophotometer (Bioteck Eon, Winooski, VT).

### Tethered Indirect Transrepression Assay

For transrepression assay, Hela cells were cultured in 5% charcoal stripped serum (CSS) DMEM media in 96‐well plates for 24 h. Then the cells were transfected with 71 ng pCMV‐GR11, 5 ng Rencilla, and 24 ng NF‐*κ*B‐Luc or 5 × AP‐1‐Luc by lip3000 transfection reagent for 24 h. After removing the medium and replaced with fresh medium, in case of NF‐*κ*B‐Luc, cells were treated with 5 ng mL^−1^ TNF‐*α* and the tested compound, and incubated for 18 h. And for AP‐1 transrepression, assay was run as above except that 1 ng mL^−1^ PMA was used to induce. Cells were lysed by the addition of 1× Passive Lysis Buffer (Promega, Cat # E1910), and luciferase activity was assayed by the Dual‐Glo Luciferase system (Promega, Cat # E1910). Data were plotted as firefly luciferase activity normalized to Renilla luciferase activity in Relative Luciferase Units.

### In Vitro GR Ligand Binding Assay

The binding of the tested compounds was assessed with the LanthaScreen TR‐FRET GR Competitive Binding Assay (Invitrogen Life Technologies, Inc.). Curves were fit using a sigmoidal dose‐response equation (variable slope) in GraphPad Prism 6.0 software. In addition, the values plotted in Figure 6b were normalized and the DMSO group is defined as 100%.

(1) $$\mathrm{Effect}\%\;=\frac{\mathrm{Ratio}\left(\mathrm{Sample}\right)}{\mathrm{Ratio}\left(\mathrm{DMSO}\right)}\;\times 100$$

### Transactivation Assay in Agonist Mode

Hela‐MMTV‐Luc reporter cells were established by transfecting plasmid pGL4.36[luc2P MMTV Hygro] into Hela cells with lip3000 transfection reagent. For generating a cell line with stable transgene expression, transfected cells were selected by co‐culture with 0.4 mg mL^−1^ Hygromycin (YEASEN, Shanghai, China). The agonist activities of the tested compounds toward GR were determined in Hela cells which were stably transfected with construct (MMTV‐Luc) by measuring the upregulation of firefly luciferase activity. Briefly, the day prior to assay, cells stably expressing MMTV‐luc were diluted and plated at 1 × 10^4^ cells per well in 96‐well white plates in DMEM medium (5% CSS). Then, cells were treated with the gradient concentrations of the tested compounds for 18 h and then luciferase activity was measured with the One‐Lumi Firefly Luciferase Assay Kit (Beyotime; #RG055M). Bioluminescence was measured with Synergy H1 (BioTek). Control wells with DMSO or DEX at 10 µm were included on each plate to define the 0% and 100% activation effects, respectively. Raw data were transformed to % effect using the following equation:

(2) $$\%\mathrm{effect}\;=\;100\times \frac{x\;-\min}{\max\;-\min}$$

### Transactivation Assay in Antagonist Mode

Similar to previous reports,^[^ ^17^ ^]^ the assay was run as above except that cells were treated by increasing the concentrations (0–50 µm) of the tested compound with 100 nm DEX. The control wells were included on each plate to define 0% inhibition (DMSO) and 100% inhibition (1 µmol L^−1^, RU486) of the DEX response.

Raw data were transformed to % inhibition using the following equation:

(3) $$\%\mathrm{inhibition}\;=\;100\times \frac{\max\;-\;x}{\max\;-\min}$$

### Quantitative Real‐Time Polymerase Chain Reaction

Raw264.7 cells were cultured in DMEM media (5% CSS) and then cultured in 6‐well plates (1 × 10^6^ cells per well). After overnight incubation, the media was removed and the cells were treated with 20 ng mL^−1^ LPS and 10 µm indicated compounds. After 18 h incubation, the total RNA was extracted using the EZ‐10 DNAaway RNA Mini‐Preps Kit according to the manufacturer's instructions (Sangon Biotech, Shanghai, China). Hifair III 1st Strand cDNA Synthesis SuperMix (YEASEN, Shanghai, China) was used to generate cDNA. Quantitative real‐time polymerase chain reactions were performed using the SYBR Green PCR Master Mix (YEASEN, Shanghai, China) kit and 0.4 µm indicated primers. Analysis of mRNA expression was performed using the Applied Biosystems QuantStudio 3. GAPDH was used as an internal control and the relative mRNA levels were analyzed by the 2^−∆∆Ct^ method. Primers used for the Q‐RT PCR analyses are following:

COX‐2 F: 5′‐TTCAAAAGAAGTGCTGGAAAAGGT‐3′

COX‐2 R: 5′‐GATCATCTCTACCTGAGTGTCTTT‐3′

GILZ F: 5′‐GCTGCACAATTTCTCCACCT‐3′

GILZ R: 5′‐GCTCACGAATCTGCTCCTTT‐3′

GAPDH F: 5′‐AGGCCGGTGCTGAGTATGTC‐3′

GAPDH R: 5′‐GCAGTTGGTGGTGCAGGATG‐3′

### Data and Statistical Analysis

All the experiments were repeated at least three times independently. The data were processed and normalized as the description above in the GR ligand binding assay and report gene assays. Graphs and results were analyzed by the software GraphPad Prism 6.0 software (San Diego, CA, USA) and results were presented as individual data points with mean ± SD. In all the statistical analyses, the differences between multiple groups were analyzed using the one‐way analysis of variance (ANOVA), followed by the Dunnett's post hoc test. *p < 0.05 was defined as significant; “ns” indicated not significance (*p* > 0.05).

## Conflict of Interest

The authors declare no conflict of interest.

## Supporting information

Supporting Information

## Data Availability

Research data are not shared.
