# Supplementary material for: Discovery of Novel GR Ligands toward Druggable GR Antagonist Conformations Identified by MD Simulations and Markov State Model Analysis
Source: Adv Sci (Weinh). 2021 Nov 26;9(3):2102435. doi: 10.1002/advs.202102435 (PMC8787434; doi:10.1002/advs.202102435)
Supplement: Supplementary file 1 — Supporting Information [file ADVS-9-2102435-s001.pdf]

ADVANCED SCIENCE
Open Access

Supporting Information

for *Adv. Sci.*, DOI: 10.1002/advs.202102435

Discovery of Novel GR Ligands towards the Druggable GR Antagonist Conformations Identified by MD Simulations and Markov State Model Analysis

Xueping Hu†, Jinping Pang†, Jintu Zhang†, Chao Shen, Xin Chai, Ercheng Wang, Haiyi Chen, Xuwen Wang, Mojie Duan, Weitao Fu, Lei Xu, Yu Kang, Dan Li, Hongguang Xia*, Tingjun Hou**

Discovery of Novel GR Ligands towards the Druggable GR Antagonist Conformations Identified by MD Simulations and Markov State Model Analysis

Xueping Hu[1,2,†], Jinping Pang[1,†], Jintu Zhang[1,†], Chao Shen[1], Xin Chai[1], Ercheng Wang[1], Haiyi Chen[1], Xuwen Wang[1], Mojie Duan[3], Weitao Fu[1], Lei Xu[4], Yu Kang[1], Dan Li[1,*], Hongguang Xia[5,*], Tingjun Hou[1,2,*]

[1] *Innovation Institute for Artificial Intelligence in Medicine of Zhejiang University, College of Pharmaceutical Sciences, Zhejiang University, Hangzhou 310058, Zhejiang, China*

[2]*State Key Lab of CAD&CG, Zhejiang University, Hangzhou 310058, Zhejiang, China*

[3]*Key Laboratory of magnetic Resonance in Biological Systems, State Key Laboratory of Magnetic Resonance and Atomic and Molecular Physics, National Center for Magnetic Resonance in Wuhan, Wuhan Institute of Physics and Mathematics, Chinese Academy of Sciences, Wuhan 430071, Hubei, China*

[4]*Institute of Bioinformatics and Medical Engineering, School of Electrical and Information Engineering, Jiangsu University of Technology, Changzhou, 213001, China*

[5]*Department of Biochemistry & Research Center of Clinical Pharmacy of The First Affiliated Hospital, Zhejiang University School of Medicine, Hangzhou 310058, Zhejiang, China*

[†]These authors contribute equally to this work.

[*]Corresponding authors

Dan Li

Email: lidancps@zju.edu.cn

Hongguang Xia

Email: hongguangxia@zju.edu.cn

Tingjun Hou

E-mail: tingjunhou@zju.edu.cn

Table S1. The conformational ensemble of AF2 induced by different ligands in the 10 μs MD simulations based on the C_α atoms RMSD of residues 567-579, 590-597 and 754-764.

Ligand	States	Populations (%, RMSD<2.5Å)	MIN_{RMSD} (Å)
apo	3H52_A	~ 0.0	2.05
	3H52_B	2.0	1.68
	3H52_C	2.0	1.15
	1M2Z	0.0	2.53
DEX	3H52_A	2.0 ↑	1.74
	3H52_B	0.5 ↓	1.95
	3H52_C	3.0 ↑	1.42
	1M2Z	~ 0.0 ↑	2.20
AZD9567	3H52_A	1.5 ↑	1.77
	3H52_B	1.5 ↓	1.90
	3H52_C	1.0 ↓	1.47
	1M2Z	~ 0.0 ↑	2.46
RU486	3H52_A	~ 0.0 –	1.95
	3H52_B	~ 0.0 ↓	2.18
	3H52_C	6.0 ↑	1.27
	1M2Z	~ 0.0 ↑	2.16

Note: active antagonist state, 3H52_A; partial active antagonist/agonist state, 3H52_B; passive antagonist state, 3H52_C; agonist state, 1M2Z.

Table S2. The chemical structures of the 88 compounds identified by structure-based virtual screening.

No.	SMILES	ChemDiv ID	MW
HP-1	c1ccccc1CC(CC)NC(=O)CSCc2c(-n3cccc3)n(nc2)-c4ccccc4	E008-0202	444.6
HP-2	Cc1cc(c(cc1)OC)NC(=O)C2CCN(CC2)S(=O)(=O)c(c3)ccc(c34)SCC(=O)N4	E959-1620	475.59
HP-3	c1cc(S(=O)(=O)N)ccc1CCNC(C2=O)CC(=O)N2c3cc(ccc3)OCC	5593-1275	417.49
HP-4	CC(C1)CCc(c12)sc(c2C(=O)N)NC(=O)c3ccc(cc3)S(=O)(=O)N(C)c4ccccc4	2513-0642	483.61
HP-5	CC(=O)Nc1ccc(cc1)NS(=O)(=O)c(c2C)cc(cc2)-c3cnc(o3)C4CC4	G408-2569	411.48
HP-6	Cc1cc(ccc1)N2CCN(CC2C)C(=O)c(c3)n(C)c(c34)c5c(n(C)c4=O)cccc5	C593-0520	428.54
HP-7	CC(=O)c1ccc(cc1)NS(=O)(=O)c(c2C)c(C)n(n2)-c(nn3)ccc3-c(cc4)ccc4C	M348-0226	461.55
HP-8	CS(=O)(=O)N(CC1)c(c12)ccc(c2)C(=O)Cn(n3)c(=O)ccc3-c4cc(OC)ccc4	Y041-3684	439.49
HP-9	Cc1c(C)ccc(c1)S(=O)(=O)N2C(CCC2=O)C(=O)N3CCN(CC3)c4cc(Cl)ccc4	G631-1649	476
HP-10	CC1CCN(CC1)C(=O)C2CCN(CC2)S(=O)(=O)c(c(C)no3)c3/C=C/c4c(F)cccc4	G637-0921	475.59
HP-11	CC(C)(C)c1ccc(cc1)S(=O)(=O)Nc(c2)c(N3CCCCC3)cc(c24)n(C)c(=O)c(=O)n4C	G266-0187	484.62
HP-12	c1ccccc1CNc(c2)c(cc(c23)n(C)c(=O)n3C)NS(=O)(=O)c(cc4)ccc4C	C301-5938	436.54
HP-13	FC(F)(F)c1cc(ccc1)N2CCN(CC2)S(=O)(=O)c(c(C)c3)cc(c34)[nH]c(=O)[nH]4	G801-0347	440.45
HP-14	c1ccccc1CC(=O)N(c(cc2)cc(c23)sc(=O)o3)S(=O)(=O)c(c4OC)cc(cc4)OC	6807-1451	485.54
HP-15	c1cccc(c12)ccc(c2)NC(=O)CN(c3cccc(C)c3C)S(=O)(=O)c4ccccc4	4577-1565	444.56
HP-16	Fc1c(Cl)cc(cc1)NC(=O)CN(C)S(=O)(=O)c(c2)ccc(c23)N(C(=O)C)CCC3	G855-4250	453.92
HP-17	Cc1cc(ccc1)N(C)C(=O)C(C)n(n2)c(=O)ccc2-c(cc3)cc(c3C)S(=O)(=O)N4CCCC4	D315-1286	494.62
HP-18	CC(C)CNC(=O)C1CCCN1C(=O)c2cc(ccc2)NS(=O)(=O)c(c3C)cc(C)cc3	L027-0277	457.6
HP-19	o1cccc1C(=O)N(CCC2)c(c23)ccc(c3)NS(=O)(=O)c(c(c4)OC)cc(C)c4C	G503-0123	440.52
HP-20	c1cccc(Cl)c1CNC(=O)C(CC(C)C)NS(=O)(=O)c(c2)ccc(c23)N(CC3)C(=O)CC	C464-0916	492.04
HP-21	c1ccccc1C(O)C(C(=O)OCC)NS(=O)(=O)c(cc2)cc3C(=O)c(c4c23)cccc4	3063-0200	451.5
HP-22	Cc1ccc(cc1)S(=O)(=O)NC(C)c2nc(no2)-c3ccc(cc3)N4CCCC4	F373-0024	412.51
HP-23	o1cccc1CNC(=O)C2(CC2)c3ccc(cc3)NS(=O)(=O)c(c4)ccc(OC)c4OC	L164-0284	570.55
HP-24	c1ccccc1C(C)CNC(=O)CC(C)S(=O)(=O)c(c2)ccc(c23)N(C(=O)C)CC3	E746-0740	428.55
HP-25	c1cc(F)cc(c1OC)S(=O)(=O)Nc(cc2)ccc2-c3ccc(nn3)N4CCCCC4	G620-0764	442.52

HP-26	c1cccc(OC)c1CN(C2)C(=O)C(C)Oc(c23)ccc(c3)NS(=O)(=O)c(cc4)ccc4F	V016-8573	470.52
HP-27	Cc1c(C)ccc(c1)NC(=O)CN(C)S(=O)(=O)c(c2)ccc(c23)[nH]c(n3)-c4ccccc4	E511-3733	448.55
HP-28	[O-]NC(=O)Cc1ccc(cc1)N(S(=O)(=O)c(c2C)cc(C)cc2)Cc3ccc(F)cc3	K783-3189	464.5
HP-29	CC(=O)c1cc(ccc1)NC(=O)C(Oc(c23)cc(C)cc2)CN3S(=O)(=O)c(cc4)ccc4C	J014-2107	464.54
HP-30	C1COCCN1C(=O)C2C(c3cc(OC)ccc3)CN(C2)S(=O)(=O)c4ccccc4	V003-4684	430.53
HP-31	c1cccc(C)c1C(C)N(S(=O)(=O)c(cc2)cc(Cl)c2F)c3cc(OC)ccc3	V016-9604	433.93
HP-32	N#Cc1c(C)c(c(C)[nH]c1=O)Cc2ccc(cc2)S(=O)(=O)N(CC3)CCN3c4ccc(F)cc4	D500-0018	480.57
HP-33	C1COCCN1C(=O)c(cccc2)c2C(=O)Nc3ccc(cc3)S(=O)(=O)N4C(C)CCCC4C	8015-0809	485.61
HP-34	c1ccccc1-n(n2C)c(=O)c(c2C)NC(=O)c3ccc(cc3)N(C)S(=O)(=O)c4ccccc4	4754-2081	476.56
HP-35	O=C1CCCN1CCCNC(=O)CCn(cc2)c(c23)ccc(c3)S(=O)(=O)N4CCCCC4	E848-1575	460.6
HP-36	c1cccc(c12)nn(n2)-c(c3C)cc(O)c(c3)NS(=O)(=O)c(c4)ccc(c45)cccc5	8012-6237	430.49
HP-37	c1ccccc1N(CC)C(=O)Cn2c(=O)oc(c23)cc(cc3)S(=O)(=O)N(C4)CC(C)CC4C	C732-0853	471.58
HP-38	c1cc(Cl)ccc1C(=O)c(c2C)oc(c23)cc(cc3)NS(=O)(=O)c(cc4)ccc4C	F265-0257	439.92
HP-39	C1CCCCCN1C(=O)CCC(=O)N(CC2)c(c23)ccc(c3)S(=O)(=O)Nc(cc4)cc(F)c4C	F325-1728	487.6
HP-40	CCc1ccc(cc1)NC(=O)CN2C(=O)COc(c23)ccc(c3)S(=O)(=O)Nc4ccccc4	E746-0459	465.53
HP-41	NC(=O)C1CCN(CC1)c2ncnc(c23)n(-c4c(OC)cccc4)cc3-c5ccccc5	D447-0452	427.51
HP-42	c1ccccc1\C=C\C(=O)NCC(=O)N/N=C\2C(=O)N(c(c23)cccc3)CCc4ccccc4	1805-1396	452.52
HP-43	c1cc(S(=O)(=O)N)ccc1-n(c(C)c2)c(C)c2/C=C3\C(C)=NN(C3=O)c4ccccc4	4742-2736	434.52
HP-44	c1ccccc1CN(C(=O)C)c(s2)nc(C)c2C(=O)NCCc3c[nH]cn3	Y041-8499	383.48
HP-45	o1cccc1C(=O)c2cn(c(c23)cccc3)CC(=O)Nc(c4C(F)(F)F)cccc4	6948-4599	412.37
HP-46	c1ccccc1CNC(=O)c2csc(n2)-c3ccc(cc3)OCc4c(F)cc(F)cc4	V018-3905	436.48
HP-47	c1ccccc1C(=O)NC(C(=O)NCc2ccco2)C3CCN(CC3)C(=O)c4ccc(C)cc4	V019-0135	459.55
HP-48	c1cccc(c12)n3c(c(C#N)c(C)cc3=O)n2CC(O)CN4CCN(CC4)c5ccccc5	D724-0206	441.54
HP-49	c1cc(F)ccc1CNS(=O)(=O)c(c2CC)cc(cc2)-c3nn(C)c(=O)c(c34)CCCC4	D505-0628	455.56
HP-50	c1ncccc1CNC(=O)c(c2=O)c[nH]c(c23)ccc(c3)S(=O)(=O)Nc(cc4C(F)(F)F)ccc4	K788-0860	502.48
HP-51	s1cccc1CN(C(C)CC)CCN(CC2=O)C(N)=C2c(n3)sc(c34)cccc4	F187-0266	426.61
HP-52	c1ccccc1N(C)CCCNC(=O)CS(=O)Cc2c(C)oc(n2)-c(c3C)cccc3	C527-1183	439.58
HP-53	C1COCCN1C(=O)c2ccc(cc2)-c(n3)oc(C)c3CS(=O)c(cc4)ccc4C	E776-1623	424.52
HP-54	c1cccc(OC)c1CNC(=O)CN2C(=O)C(C)=C(S2(=O)=O)c(c3)ccc(C)c3C	G206-0092	428.51

HP-55	CN(C)C(=O)c1ccc(cc1)Nc(c(c23)cccc3)nnc2-c(cc4)cc(c4C)S(=O)(=O)NC	3938-1463	475.57
HP-56	c1cccc(c12)CN(CC2)C(=O)Cc3csc(n3)NS(=O)(=O)c4ccc(Cl)cc4	D715-1334	447.97
HP-57	c1ccc(F)cc1CNC(=O)CC2N(CCc(c23)cccc3)S(=O)(=O)c(cc4)ccc4NC(=O)C	F714-0880	495.58
HP-58	c1cccc(c12)oc(cc2=O)C(=O)N(C(CC3)CS3(=O)=O)Cc4ccc(F)cc4	D103-2362	415.44
HP-59	c1ccccc1NC(=O)Cn(c(c23)ccc(C)c3)cc(c2=O)S(=O)(=O)c(cc4)ccc4OC	C655-0349	462.53
HP-60	c1c(Cl)ccc(OC)c1NC(=O)CN(C(=N2)SC(C23)CS(=O)(=O)C3)c(c4)cc(C)cc4C	C998-0210	494.04
HP-61	CCC(=O)N(CC1)c(c12)ccc(c2)S(=O)(=O)CCC(=O)N3CCN(CC3)c4c(Cl)cccc4	C361-2029	490.03
HP-62	c1cc(C)ccc1Cn(c(c23)cc(cc3)OC)c(C(=O)O)c2CNCCc4ccc(cc4)SC	E518-0934	474.63
HP-63	o1cccc1C(=O)Nc(ccc2)cc2C(=O)N(CC3)CCC3NS(=O)(=O)c(cc4)ccc4C	L291-0190	467.55
HP-64	CCC(=O)Nc1cc(ccc1)Oc(c2)ccc(c23)C(=O)N(C3=O)c4ccc(cc4)OC	3226-0121	416.44
HP-65	C1CCCCC1NC(=O)c2c(cccc2)NC(=O)c(c3)ccc(c34)N(S(=O)(=O)C)CCC4	8018-5472	455.58
HP-66	c1ccccc1CNC(=O)CSc(nn2)ccc2-c(c3C)sc(n3)-c4cccs4	G954-0175	438.6
HP-67	c1c(F)ccc(c1C)S(=O)(=O)Nc(c2)ccc(c23)N(CCC3)S(=O)(=O)c(cc4)ccc4C	G505-0106	474.58
HP-68	c1cccc(c12)CN(CC2)C(c3ccc(cc3)N(C)C)CNS(=O)(=O)CCc4ccccc4	G501-0462	463.65
HP-69	c1ccccc1CC(C(=O)Nc(cc2C(F)(F)F)ccc2)NS(=O)(=O)c(c3)ccc(c34)NC(=O)CC4	C597-0015	517.53
HP-70	CC(C)(C)c1ccc(cc1)C(=O)Nc(n(n2)-c3ccccc3)cc2-c4c(F)cccc4	G771-0723	413.5
HP-71	c1ccccc1C(=O)Nc(sc(C)c2)c2C(c3ccc(cc3)SC)Nc4ccccn4	D444-0139	445.61
HP-72	c1cc(F)ccc1-n(n2)ccc(=O)c2C(=O)Nc3ccc(cc3)S(=O)(=O)N(CC)c4ccccc4	D223-1013	492.53
HP-73	Cc1ccc(cc1)S(=O)(=O)CC(=O)Nc(c2C#N)sc(c23)CN(CC3)Cc4ccccc4	G856-7191	465.6
HP-74	c1cc(F)ccc1N2CC(CC2=O)C(=O)Nc3ccc(cc3)S(=O)(=O)NCCc4ccccc4	Y031-2935	481.55
HP-75	O=S1(=O)CC(C)C(=O)N1c(cc2)ccc2C(=O)Nc3c(cccc3)Oc4ccccc4	D473-1078	436.49
HP-76	CCOC(=O)C1CCCN(C1)C(=O)Cc2ccc(cc2)N(C(=O)C3CC3)Cc4ccc(F)cc4	V028-9004	466.56
HP-77	s1cccc1CNC(=O)C(Cc2ccccc2)NS(=O)(=O)c(ccc3)c(c34)nccc4	G319-0160	451.57
HP-78	CCN(CC)c(c1)ccc(c1C)NC(=O)CCNS(=O)(=O)c(c2)ccc(c23)NC(=O)CC3	C464-3208	458.58
HP-79	NC(=O)COc(cc1)c(OC)cc1C(CC(=O)N2)c(c23)scc3S(=O)(=O)c(cc4)ccc4C	D563-0162	486.57
HP-80	CC(=O)N(CC1)CCN1C(=O)Cc2ccc(cc2)N(C(=O)c3ccccc3)Cc4cc(C)ccc4	V016-9187	469.59
HP-81	Cc1ccc(cc1)S(=O)(=O)c(cc2)c(NC(=O)C)cc2C(=O)N3CCN(CC3)c4cccc(C)c4C	K786-9415	505.64
HP-82	c1ccccc1CN(C(=S)S2)C(=O)/C2=C\c3ccc(o3)-c4ccc(cc4)S(=O)(=O)N	0423-0163	456.56
HP-83	Cc1c(C)cc(c(C)c1)S(=O)(=O)Nc(ccc2)cc2-c(c3)[nH]c(c34)cccc4	F788-0145	390.51

HP-84	c1cccc(C)c1C(=O)NC(C(=O)N2CCCC2)C3CCN(CC3)C(=O)CCc4ccccc4	V015-6088	461.61
HP-85	c1ccccc1C(C)CNS(=O)(=O)c(cc2)ccc2CCN(C3=O)C(=O)c(c34)nccc4	E734-2528	449.53
HP-86	c1ccccc1C(O)(c2ccccc2)C3CCN(CC3)C(=S)Nc4ccc(cc4)S(=O)(=O)N	6208-0881	481.64
HP-87	COC(=O)c1c(cccc1)NC(=O)CSc(c2S(=O)(=O)c3ccccc3)[nH]c(n2)-c4ccccc4	D406-0335	507.59
HP-88	COC(=O)c1c(cccc1)NC(=O)CN(S(=O)(=O)C)c(c(Cl)cc2)cc2C(F)(F)F	4577-1365	464.85

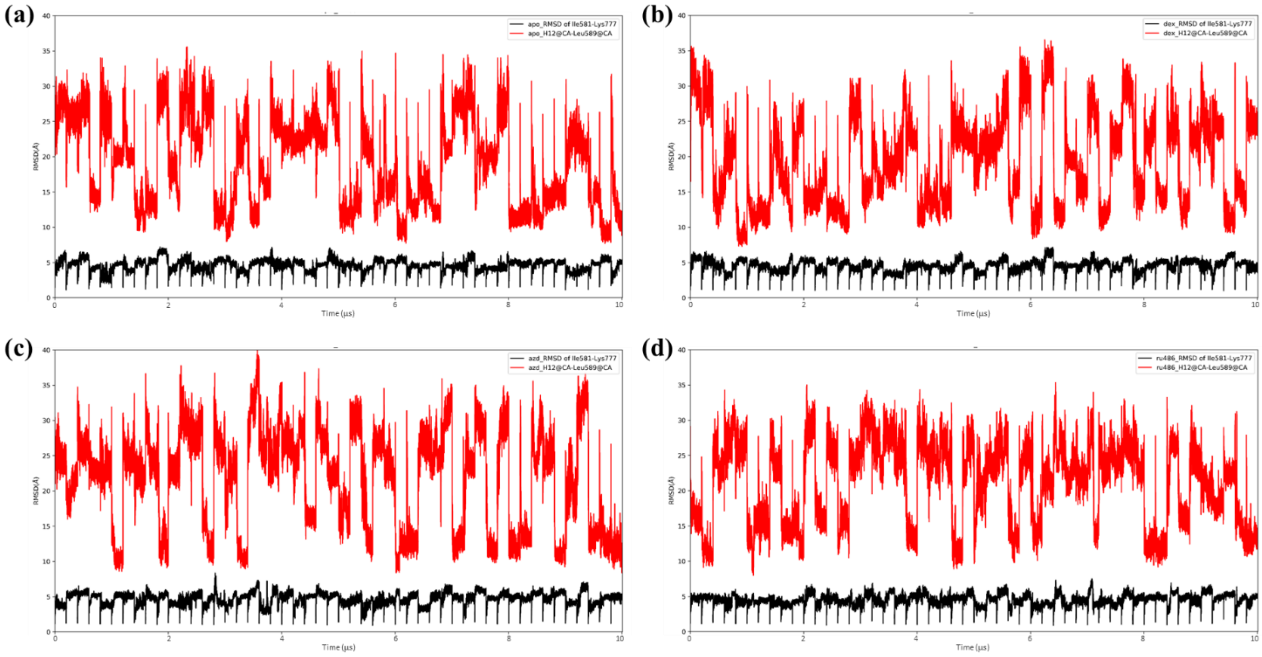

Figure S1. The evolution of the RMSD of Ile581-Lys777, and the distance between H12 (Ala754-Tyr764) and Leu589 over time for (a) apo-LBD, (b) dex-LBD, (c) azd-LBD, and (d) ru486-LBD.

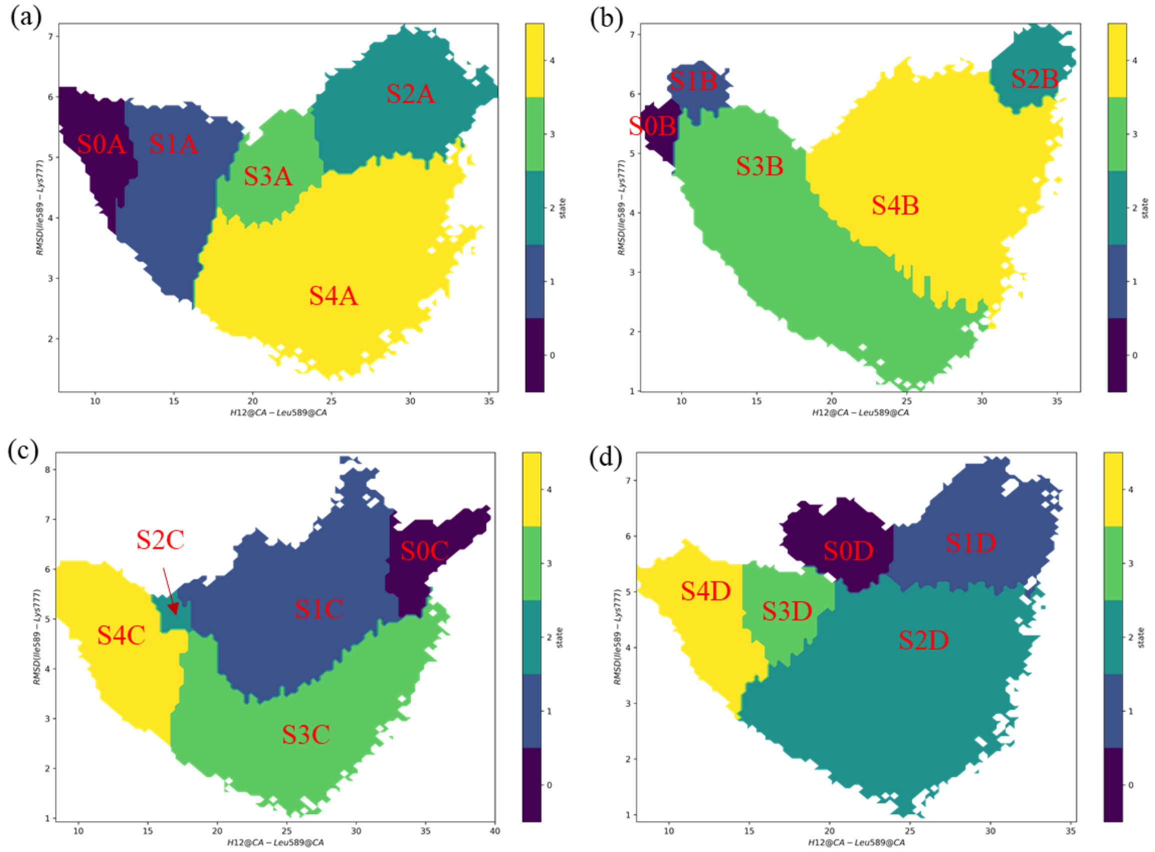

Figure S2. The five metastable macrostates calculated by PCCA++ for apo-LBD (a), dex-LBD (b), azd-LBD (c), and ru486-LBD (d).

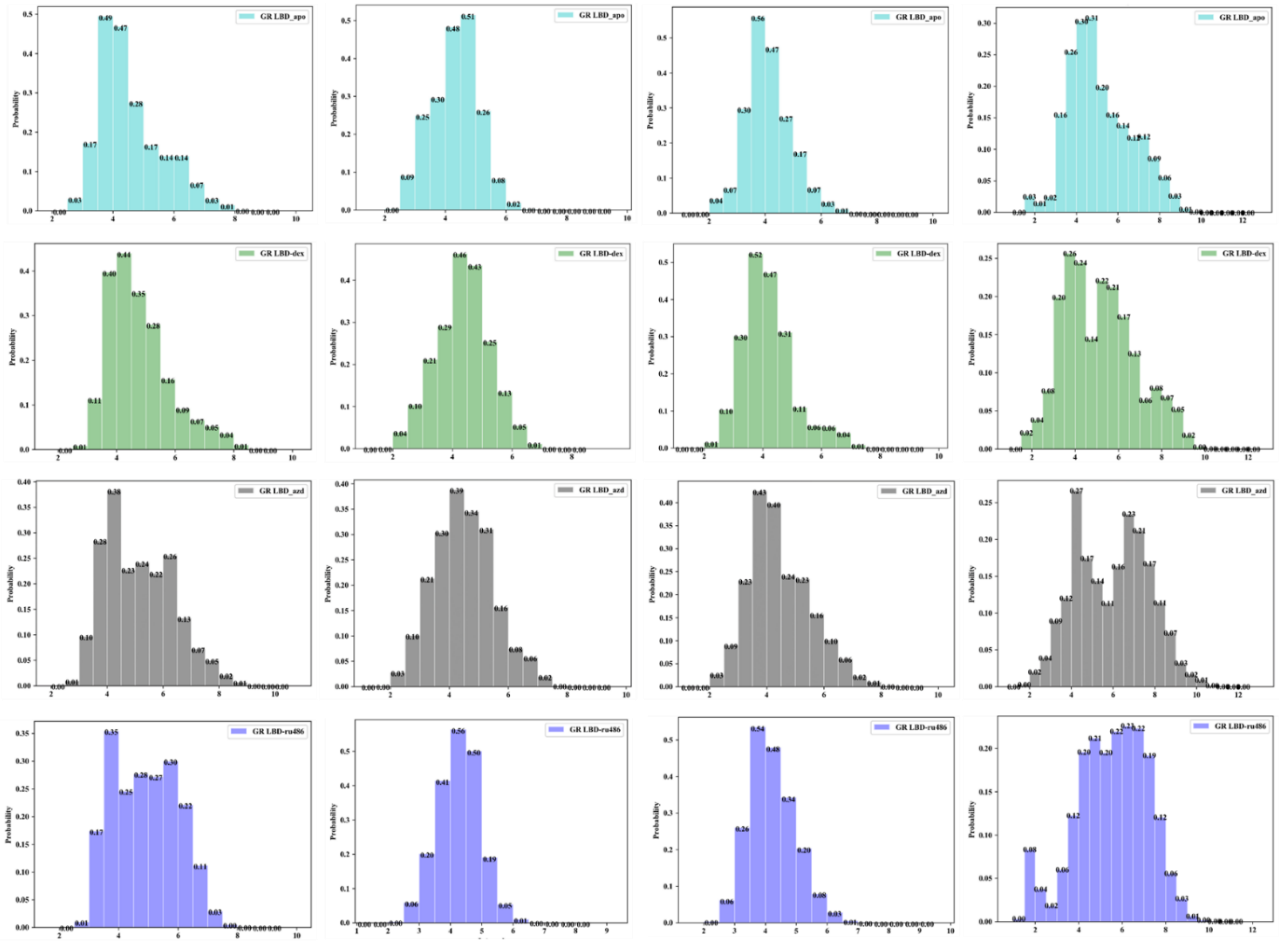

1M2Z AF2 sampling 3H52_A AF2 sampling 3H52_B AF2 sampling 3H52_C AF2 sampling

Figure S3. The population distributions of the four experimental AF2 conformations (3H52_A, 3H52_B 3H52_C, and 1M2Z.) in the four systems (apo-LBD, dex-LBD, azd-LBD, and ru486-LBD). The RMSD of the AF2 C_α atoms (including the residues 567-579, 590-597 and 754-764) was used as the criterion to compare the similarity between the conformation from the 10 μs MD simulation trajectories and the experimental structure.

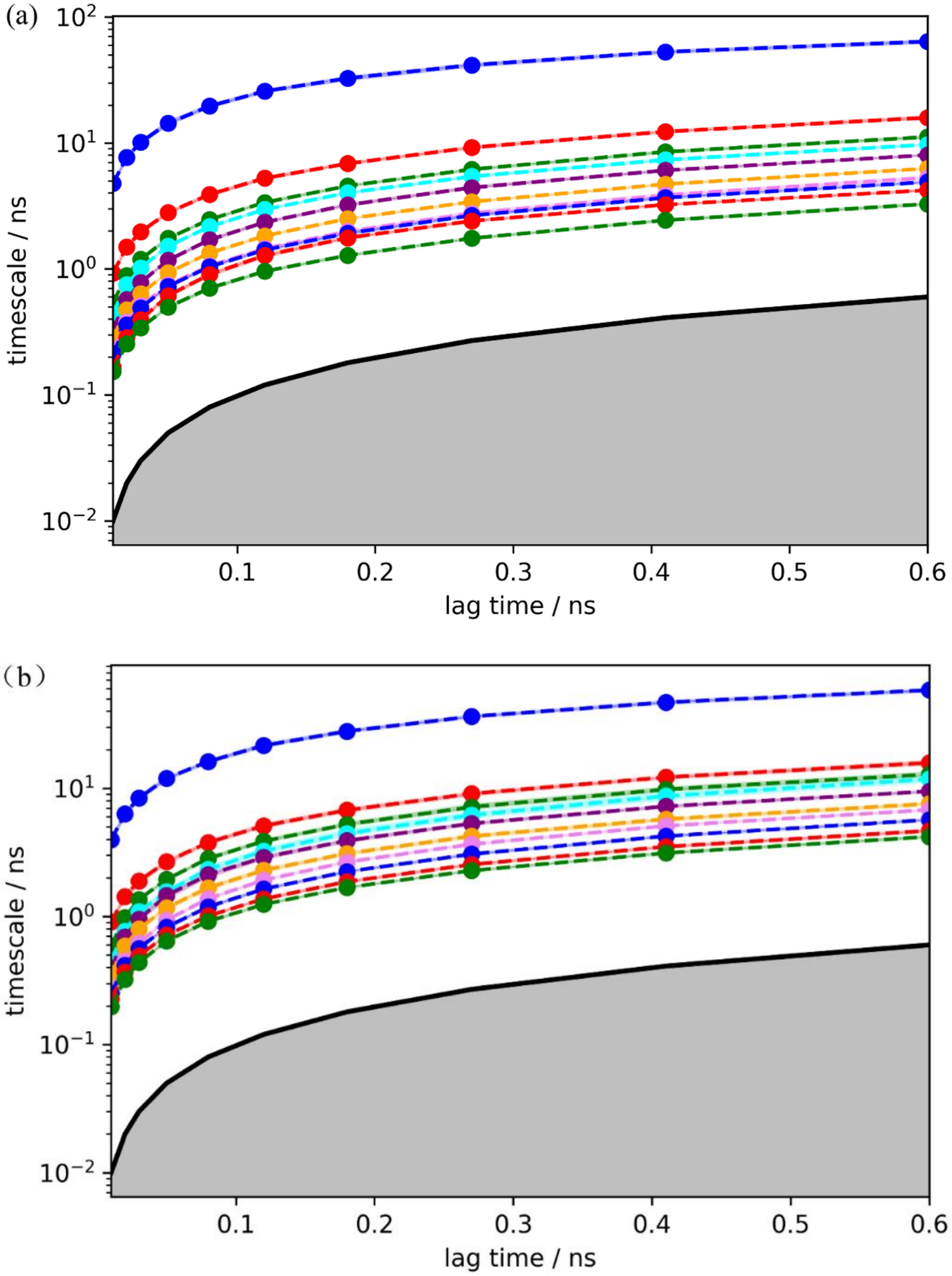
(a)
timescale / ns
lag time / ns
(b)
timescale / ns
lag time / ns

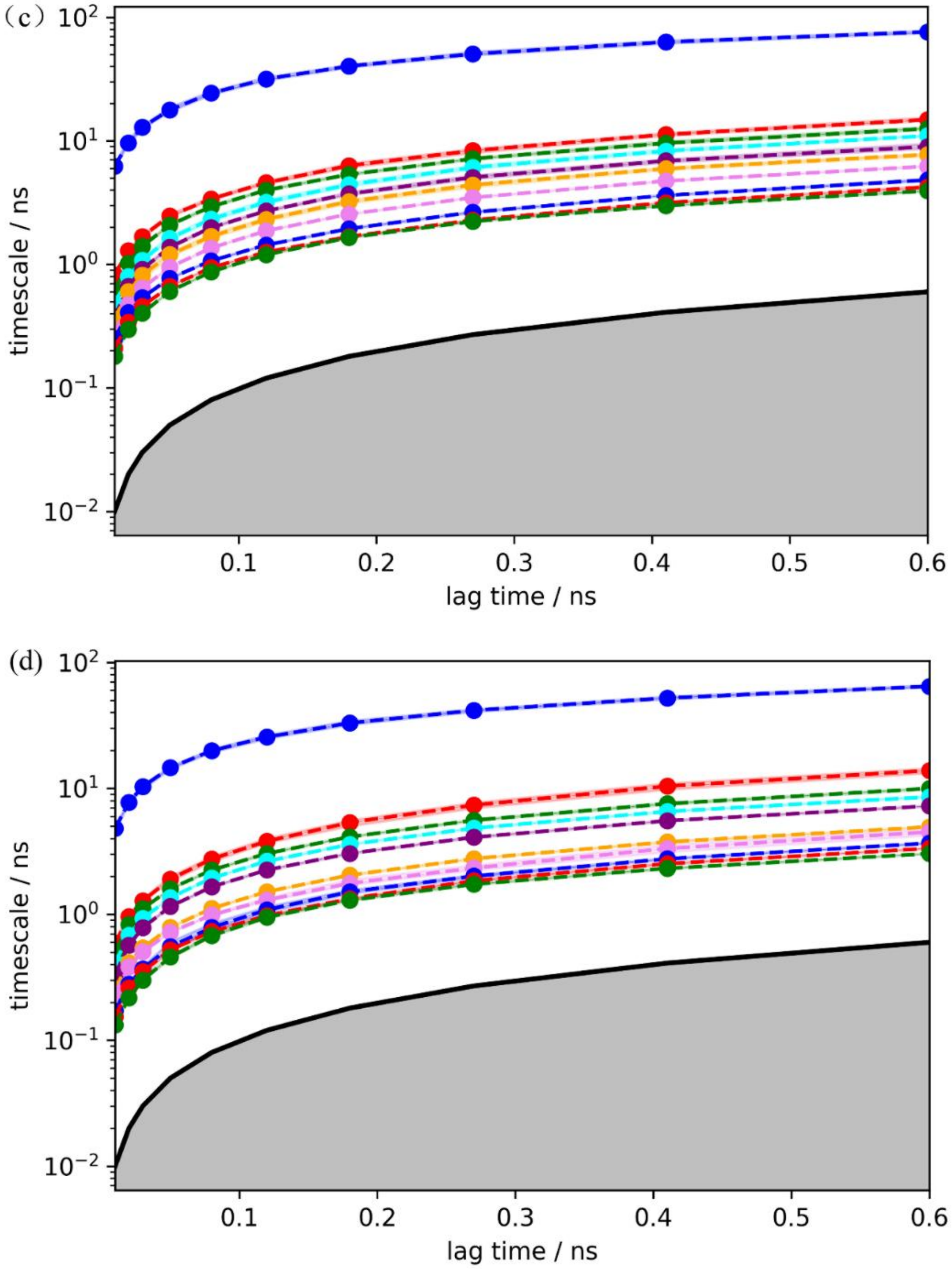

Figure S4. The implied timescales versus lag time for the (a) apo-LBD, (b) dex-LBD, (c) azd-LBD, and (d) ru486-LBD systems. The implied timescales of a dynamic system will tend to be constant with the increasing of the lag time if the system satisfies the Markov State Model. When estimating the Markov State Model, the smallest lag time was used to obtain the model with highest time resolution. Thus, we chose 20 steps (0.2 ns) as the lag time.

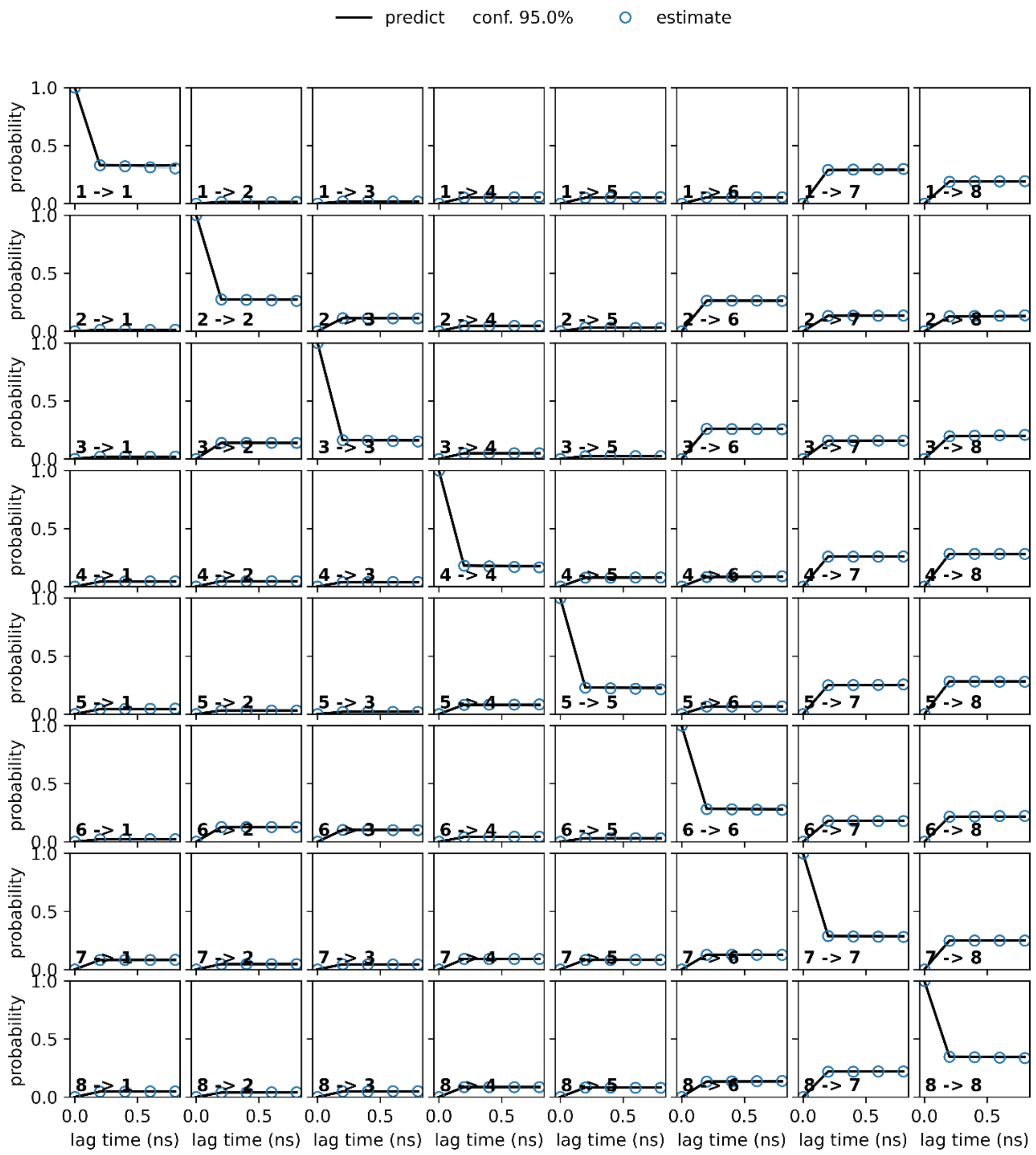

Figure S5. Generalized Chapman-Kolmogorov tests for the eight microstates of the apo-LBD system. We compare the estimated transition probabilities calculated from the MD data (circles) and the predictions of the MSMs with different lag times. For the states 1 to 8, a nearly perfect agreement were observed for all the lag times, indicating the high Markovianity of the microstates.

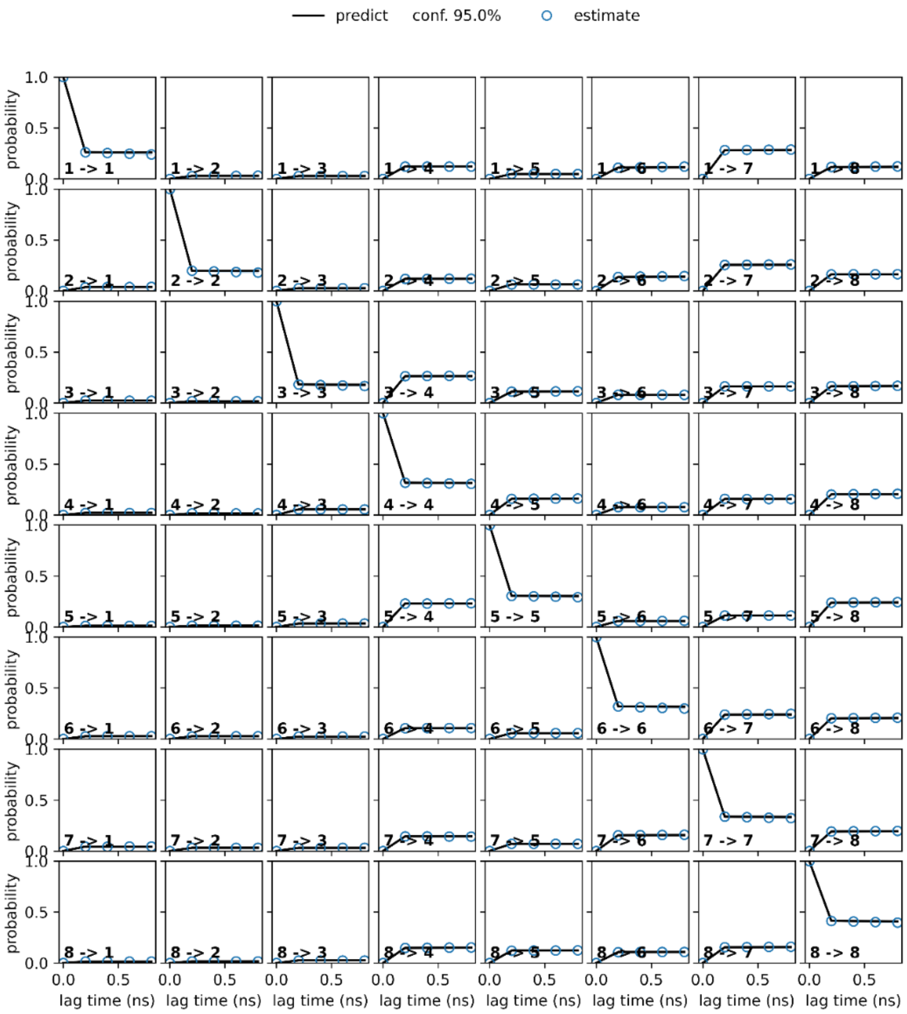

Figure S6. Generalized Chapman-Kolmogorov tests for the eight microstates of the dex-LBD system.

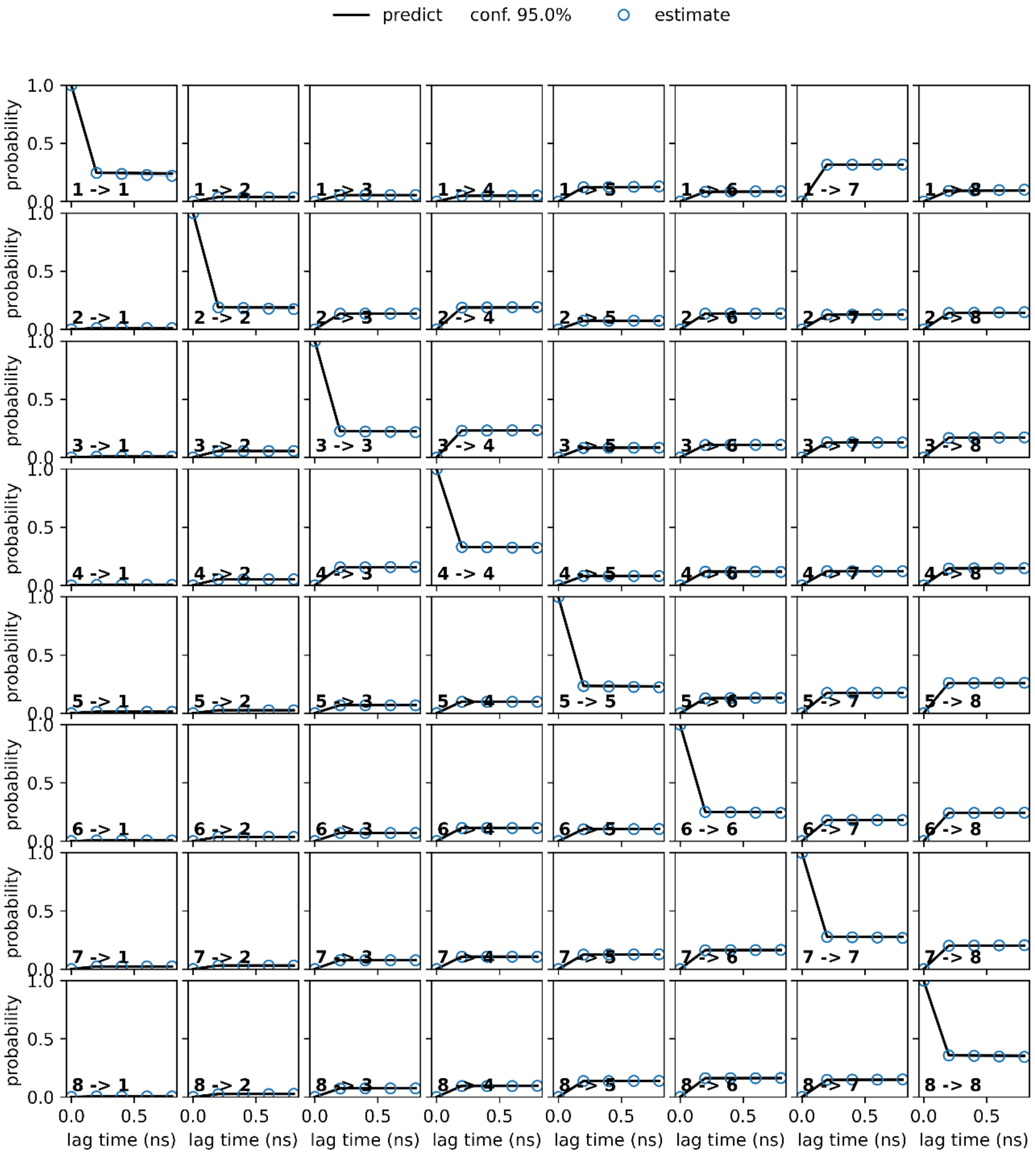

Figure S7. Generalized Chapman-Kolmogorov tests for the eight microstates of the azd-LBD system.

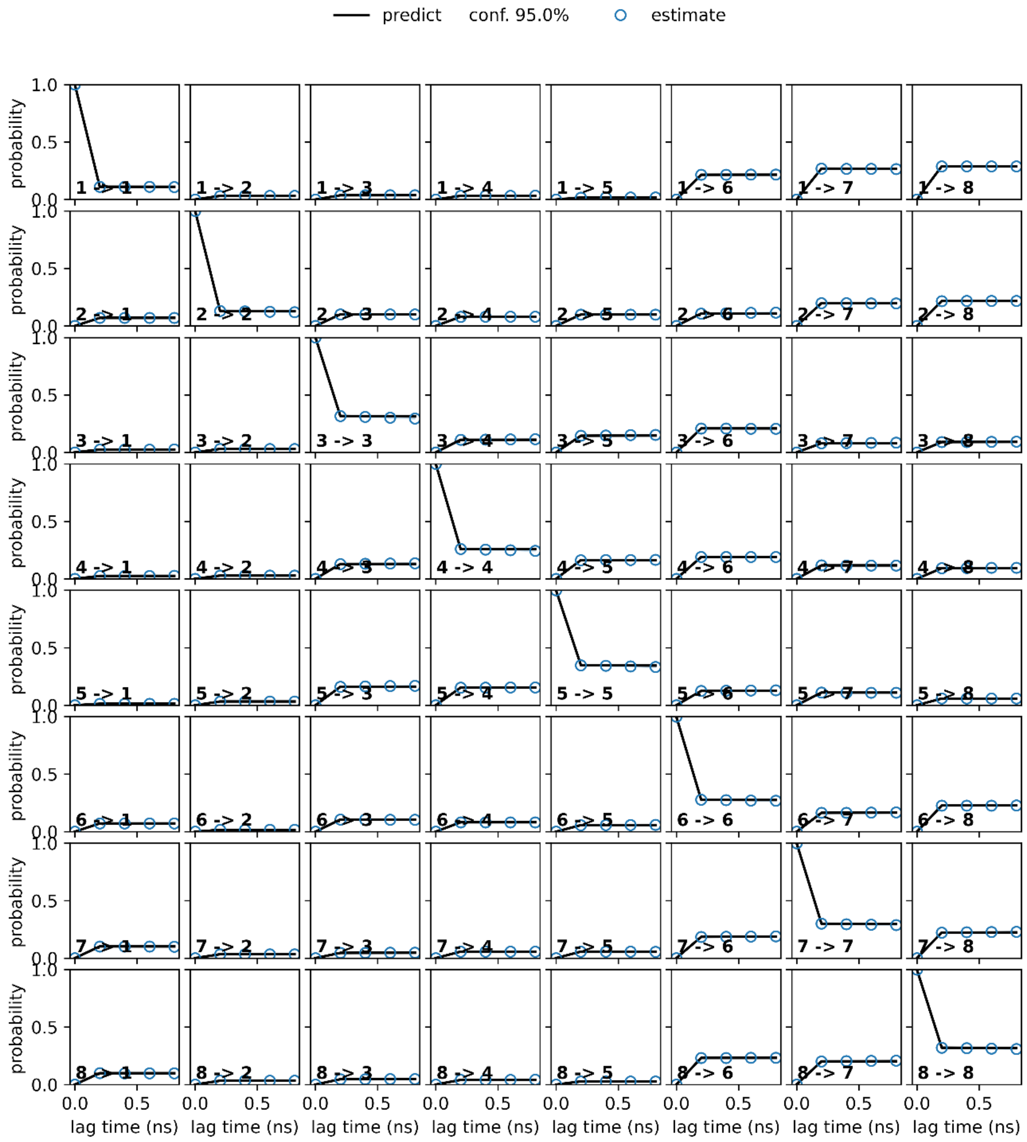

Figure S8. Generalized Chapman-Kolmogorov tests for the eight microstates of the ru486-LBD system.

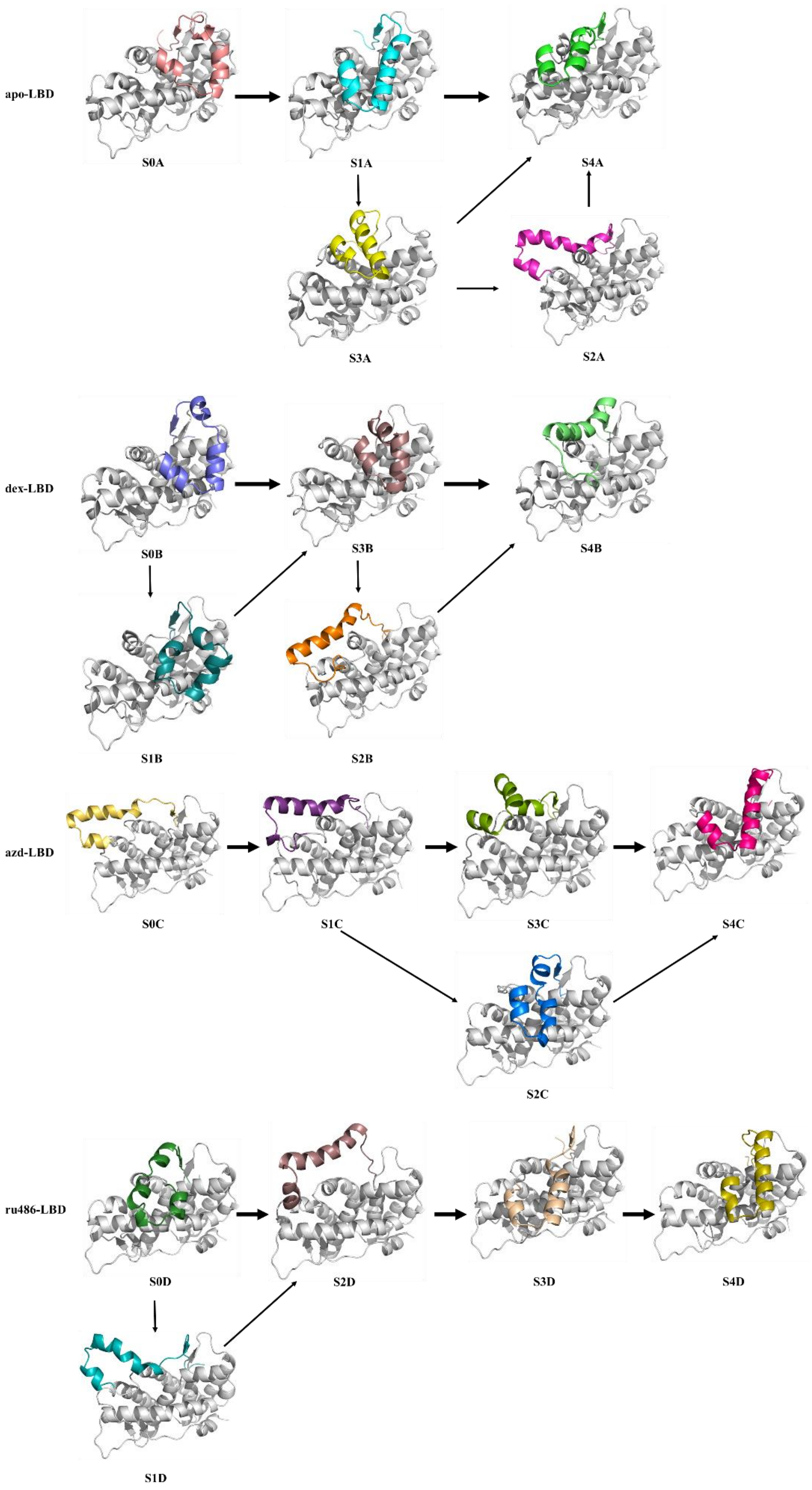

Figure S9. The structures of the main pathways in (a) apo-GR(a), (b) dex-LBD(b), (c) azd-LBD and (d) ru486-LBD.

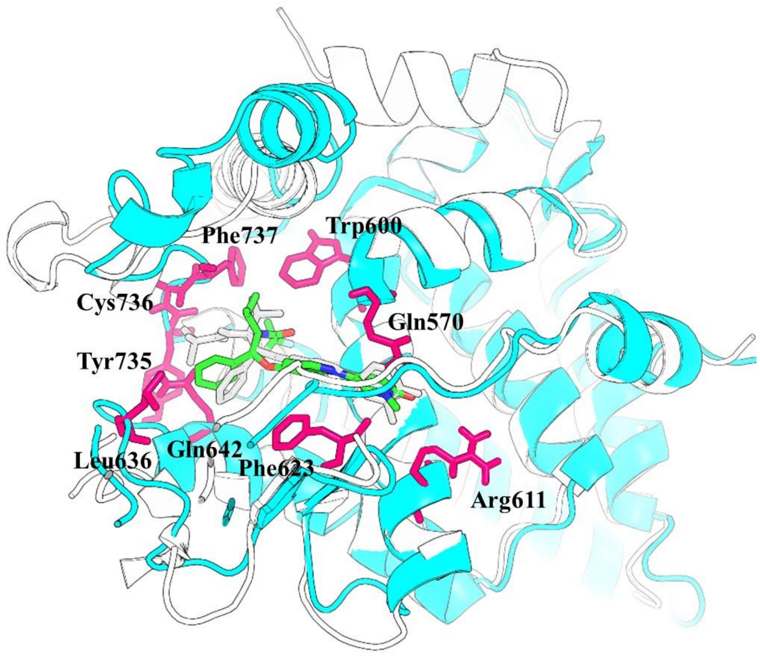

Figure S10. The passive antagonist states (in cyan) in the in the azd-LBD system. AZD9567 in the passive antagonist states was shown in green. The RMSD was calculated using the CA atoms of AF2. The structure of PDB 6EL9 was shown in gray.

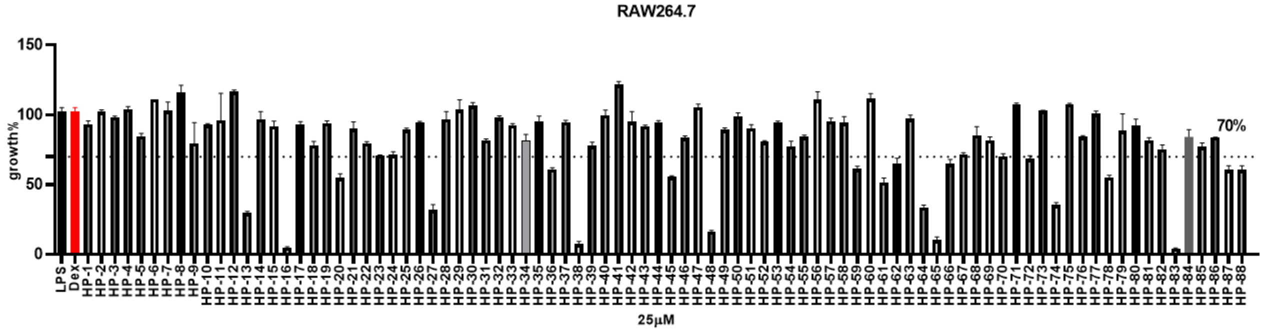

Figure S11. inhibition percentage of cell viability in HeLa cell lines treated with 25 μM tested compounds for 48 h.

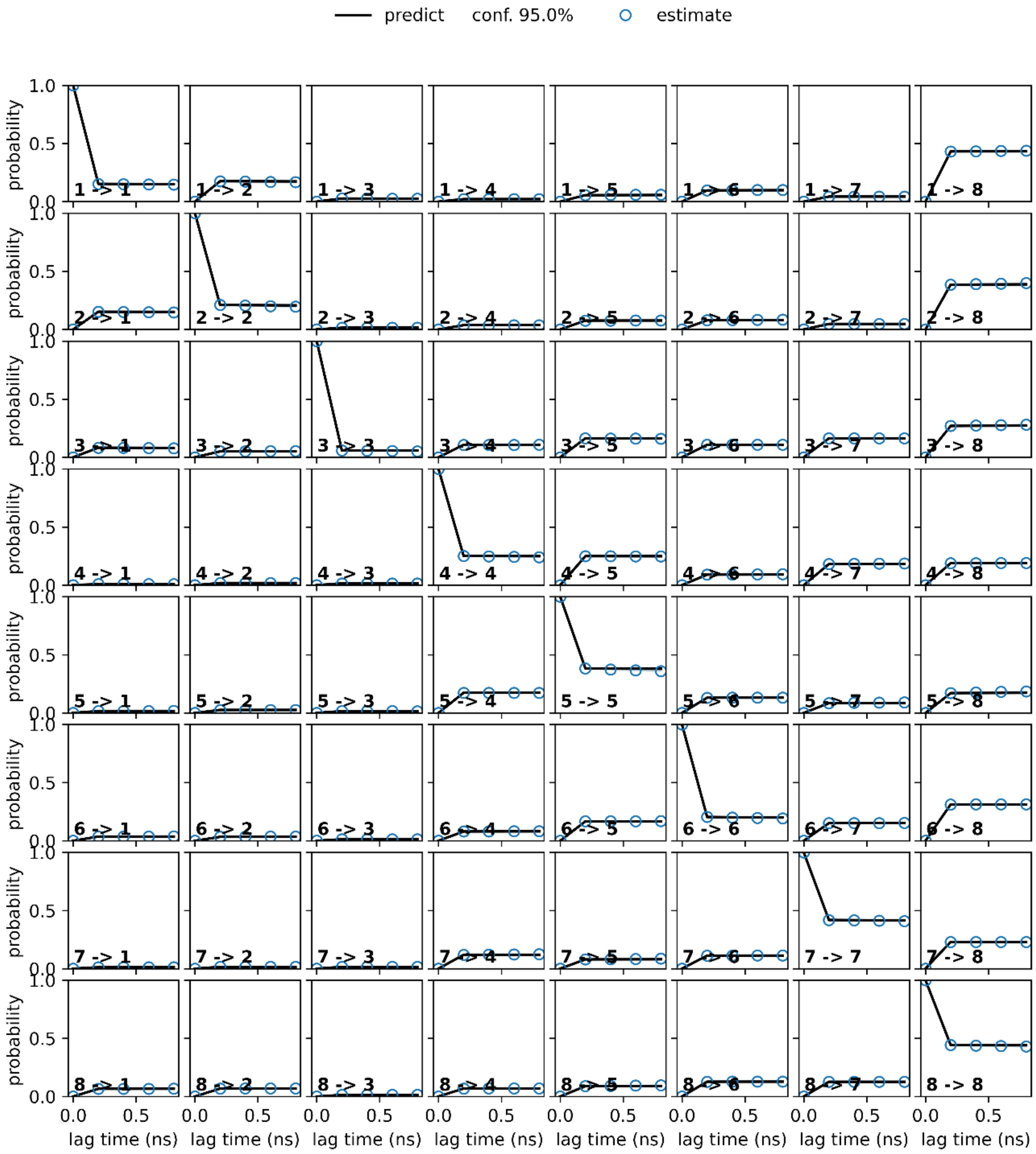

Figure S12. Generalized Chapman-Kolmogorov tests for the eight microstates of the HP19-LBD system.
